# Discrimination of β-cyclodextrin/hazelnut (*Corylus avellana* L.) oil/flavonoid glycoside and flavonolignan ternary complexes by Fourier-transform infrared spectroscopy coupled with principal component analysis

**DOI:** 10.3762/bjoc.19.30

**Published:** 2023-03-28

**Authors:** Nicoleta G Hădărugă, Gabriela Popescu, Dina Gligor (Pane), Cristina L Mitroi, Sorin M Stanciu, Daniel Ioan Hădărugă

**Affiliations:** 1 Doctoral School “Engineering of Vegetable and Animal Resources”, University of Life Sciences “King Mihai I” from Timişoara, Calea Aradului 119, 300645 Timişoara, Romania; 2 Research Institute for Biosecurity and Bioengineering, Calea Aradului 119, 300645 Timişoara, Romania; 3 Department of Food Science, University of Life Sciences “King Mihai I” from Timişoara, Calea Aradului 119, 300645 Timişoara, Romania; 4 Department of Rural Management and Development, University of Life Sciences “King Mihai I” from Timişoara, Calea Aradului 119, 300645 Timişoara, Romania; 5 Department of Economy and Company Financing, University of Life Sciences “King Mihai I” from Timişoara, Calea Aradului 119, 300645 Timişoara, Romania; 6 Department of Applied Chemistry, Organic and Natural Compounds Engineering, Polytechnic University of Timişoara, Carol Telbisz 6, 30001 Timişoara, Romania

**Keywords:** antioxidant, cyclodextrin, flavonoid, hazelnut vegetable oil, ternary supramolecular inclusion complex

## Abstract

The goal of the study was the discrimination of β-cyclodextrin (β-CD)/hazelnut (*Corylus avellana* L.) oil/antioxidant ternary complexes through Fourier-transform infrared spectroscopy coupled with principal component analysis (FTIR–PCA). These innovative complexes combine the characteristics of the three components and improve the properties of the resulting material such as the onsite protection against oxidative degradation of hazelnut oil unsaturated fatty acid glycerides. Also, the apparent water solubility and bioaccessibility of the hazelnut oil components and antioxidants can be increased, as well as the controlled release of bioactive compounds (fatty acid glycerides and antioxidant flavonoids, namely hesperidin, naringin, rutin, and silymarin). The appropriate method for obtaining the ternary complexes was kneading the components at various molar ratios (1:1:1 and 3:1:1 for β-CD hydrate:hazelnut oil (average molar mass of 900 g/mol):flavonoid). The recovering yields of the ternary complexes were in the range of 51.5–85.3% and were generally higher for the 3:1:1 samples. The thermal stability was evaluated by thermogravimetry and differential scanning calorimetry. Discrimination of the ternary complexes was easily performed through the FTIR–PCA coupled method, especially based on the stretching vibrations of CO groups in flavonoids and/or CO/CC groups in the ternary complexes at 1014.6 (± 3.8) and 1023.2 (± 1.1) cm^−1^ along the second PCA component (PC_2_), respectively. The wavenumbers were more appropriate for discrimination than the corresponding intensities of the specific FTIR bands. On the other hand, ternary complexes were clearly distinguishable from the starting β-CD hydrate along the first component (PC_1_) by all FTIR band intensities and along PC_2_ by the wavenumber of the asymmetric stretching vibrations of the CH groups at 2922.9 (± 0.4) cm^−1^ for ternary complexes and 2924.8 (± 1.4) cm^−1^ for β-CD hydrate. The first two PCA components explain 70.38% from the variance of the FTIR data (from a total number of 26 variables). Other valuable classifications were obtained for the antioxidant flavonoids, with a high similarity for hesperidin and naringin, according to FTIR–PCA, as well as for ternary complexes depending on molar ratios. The FTIR–PCA coupled technique is a fast, nondestructive and cheap method for the evaluation of quality and similarity/characteristics of these new types of cyclodextrin-based ternary complexes having enhanced properties and stability.

## Introduction

Cyclodextrins (CDs) are studied for more than one hundred years due to their unique properties related to their spatial macrocyclic structure that comprises six to eight α-ᴅ-glucopyranose (Glc*p*) units for the natural α-, β-, and γ-CD [1–3]. All hydroxy groups are oriented to the exterior of the macrocycle, leading to high water solubility. On the other hand, the tetrahydropyran moieties of the Glc*p* units provide the hydrophobic property of the CD cavity [4]. As a consequence of their unique structure, CDs can encapsulate hydrophobic molecules or hydrophobic moieties of geometrically compatible bioactive compounds [5]. The resulting supramolecular inclusion complexes provide enhanced water solubility and bioavailability/bioaccessibility of the nanoencapsulated bioactive compounds, higher oxidative and thermal stability or photostability of labile compounds, and their controlled release [6–7].

Vegetable oil and animal fat components that especially consist of fatty acid (FA) triglycerides are appropriate guest molecules for obtaining CD-based complexes. The hydrophobic long-chain moieties of the FA glycerides allow obtaining CD:FA glyceride complexes at various molar ratios [8–9], with increased apparent water solubility and bioaccessibility of the oil and fat components. The oxidative stability of the polyunsaturated FA glycerides or free FAs is significantly increased by CD nanoencapsulation. Thus, a high thermal stability was obtained for linoleic acid encapsulated into α-CD by co-crystallization [10]. Omega-3 FA glycerides such as eicosapentaenoic and docosahexaenoic acid glycerides (EPA and DHA glycerides) from fish oil are less stable against oxidation. Their thermal and oxidative stabilities were significantly increased by CD nanoencapsulation as was shown for fish oil from common barbel (*Barbus barbus* L.), Pontic shad (*Alosa immaculata* Bennett), European wels catfish (*Silurus glanis* L.), common bleak (*Alburnus alburnus* L.), common nase (*Chondrostoma nasus* L.), Atlantic salmon (*Salmo salar* L.), and European anchovy (*Engraulis encrasicolus* L.) [11–14]. The stability and the level of degradation compounds were determined by thermal methods (thermogravimetry-differential thermogravimetry, TG–DTG, and differential scanning calorimetry, DSC) and gas chromatography–mass spectrometry (GC–MS), respectively. The addition of sodium caseinate during the CD complexation of fish oils was reported to further increase the oxidation stability and retardation of odor [15]. Poultry lipids have high contents of mono- and polyunsaturated FA glycerides, especially oleic and linoleic acid glycerides. The stability of chicken lipids was significantly increased by β-CD complexation which was demonstrated by both thermal (TG–DTG and DSC) and chromatographic (GC–MS for the degradation compounds, i.e., aldehydes, formylated carboxylic acids, or dicarboxylic acids) methods [16]. Also, vegetable oils containing unsaturated FA moieties were stabilized by CD complexation. Common bean (*Phaseolus vulgaris* L.) oil contains 55.7–58.8% of polyunsaturated FAs (relative content as methyl esters), with an important fraction of omega-3 α-linolenic acid (ALA) of 14.1–18.9%. It was stabilized by β-CD complexation, with an increased content of the omega-3 FAs into the nanoencapsulated oil of >14% [17]. Other complexes between CDs and various vegetable oils have been obtained and characterized. Soybean oil was combined with α-CD for obtaining a stable dry emulsion, which implied the partial molecular encapsulation of the soybean oil triglycerides. This emulsion was prepared in order to modulate the release of indomethacin in rats. Similar α-CD-based emulsions were obtained using wheat germ, sweet almond, borage, and virgin coconut oils [9,18–19]. The stability and bioavailability of peony (*Paeonia suffruticosa* Andr.) seed oil were significantly enhanced by complexation with β-CD through the co-precipitation from a saturated solution. The peony oil content in the complex was almost 26%, with a high ratio of unsaturated FA glycerides of ≈90% [20]. In a very recent study, perilla (*Perilla frutescens* (L.) Britton) seed oil was complexed by γ-CD and the inclusion complex was used for improving the bioavailability of ALA. This omega-3 FA was found in significantly higher concentrations in the plasma of rats fed with this complex [21]. Some vegetable oils were also encapsulated using combined matrices or polymers containing CDs as was demonstrated for example for kenaf (*Hibiscus cannabinus* L.) seed oil or “Persian lilac” (*Melia azedarach* L.) seed oil. The oils were complexed by spray drying using β-CD/gum arabic/sodium caseinate or a β-CD polymer, respectively [22–23]. However, there are less studies on the CD encapsulation of non-volatile vegetable oils in comparison with essential oils. Essential oil components are also compatible guests for CD nanoencapsulation. They were studied as “pure” compounds or as essential oil mixtures (e.g., linalool, nerolidol, nootkatone, or sweet basil – *Ocimum basilicum* L., caraway – *Carum carvi* L., coriander – *Coriandrum sativum* L., fennel – *Foeniculum vulgare* Mill., dill – *Anethum graveolens* L., garlic – *Allium sativum* L., juniper – *Juniperus communis* L., clove – *Syzygium aromaticum* (L.) Merr. & L.M., and perilla – *Perilla frutescens* (L.) Britton essential oils, respectively) [24–30].

Among vegetable oils, hazelnut (*Corylus avellana* L.) oil is a valuable source of oleic acid bound in various triglyceride combinations. The highest content was observed for triolein, OOO (61–77.5% relative concentration), but also OOL (glyceryl 1,2-dioleate 3-linoleate) and OOP (glyceryl 1,2-dioleate 3-palmitate) were found in high relative contents of 10.5–22.8% and 6.4–11.0%, respectively [31]. The fatty acid profile of hazelnut oil revealed a significantly high content of oleic acid (as methyl ester, determined by GC–MS) of 74.2–82.8%, among linoleic acid and even ALA (9.8–18.7% and ≈0.1%, respectively) [32–33]. The very high content of unsaturated fatty acid glycerides significantly decreases the stability of hazelnut oil. Only one study was performed on the γ-CD nanoencapsulation of hazelnut oil by a co-precipitation method and the thermal decomposition of the complex was evaluated by TG [34].

One way of enhancing the oxidative stability of oils and fats is the addition of antioxidants. Among food grade antioxidants, natural polyphenols such as flavonoids and flavonoid-based extracts are widely used [35–41]. Generally, flavonoids have a high number of phenolic hydroxy groups that provide the antioxidant activity. On the contrary, the presence of highly hydrophilic groups such as saccharide moieties in flavonoid glycosides reduces the level of hydrophobic interactions with the CD cavity. However, less hydrophilic moieties of flavonoid glycosides or flavonolignans interact with CDs (i.e., 4-hydroxyphenyl, 3,4-dihydroxyphenyl- and 3-methoxy-4-hydroxyphenyl moieties in the hesperidin, naringin, and rutin aglycones or silibinin). There are many studies revealing the interaction of flavonoids, flavonoid glycosides, and flavonolignans with CDs, especially for obtaining binary complexes [42–49].

In a ternary complex, considering the vegetable oil as a single component, an on-site antioxidant can protect labile FA glycerides by co-nanoencapsulation into a CD cavity. However, it is very difficult to evaluate the way of interaction in such multicomponent systems. There are some studies on the CD-based ternary complexes, but they do not deal with triglyceride-based vegetable oils or with flavonoid glycosides/flavonolignans. Most of these studies are related to controlled release of various drugs from the CD complexes such as diosmin and polyethylene glycol, haloperidol and lactic acid, cyclosporine A and polyvinyl alcohol, ketoprofen and phospholipids, dihydroartemisinin and lecithin, cefixime and ʟ-arginine, flurbiprofen and naproxen/ketoprofen/ethenzamide [50–59].

Fourier-transform infrared spectroscopy (FTIR) is a very fast, nondestructive and cheap method suitable for the evaluation of such ternary complexes. The coupling of FTIR or other spectroscopic or chromatographic techniques with a multivariate statistical analysis method (e.g., principal component analysis, PCA) allows the evaluation of the similarity/dissimilarity of complexes, as well as the identification of the variables that have significance for these classifications. FTIR–PCA was successfully applied for the discrimination of raw and thermally processed chicken lipid/β-CD complexes [16]. Moreover, raw and recrystallized β-CD samples (from water and alcohol–water solutions) were successfully classified by the FTIR–PCA technique [4]. In other studies, PCA was coupled with GC–MS for the classification of β-CD/*Ocimum basilicum* L. essential oil complexes and raw and thermally processed Mangalitza (*Sus scrofa domesticus*) lipid fractions, as well as for the discrimination of organic apples (*Malus domestica* Borkh.) on the basis of antioxidant properties and radical scavenging kinetics [27,60–61]. However, only few studies have been published on the discrimination of CD-based complexes using multivariate statistical analysis. They are especially related to the retention behavior of various biologically active molecules on CD-coated polymers used in chromatography [62]. PCA was used for the evaluation of the similarity/dissimilarity of some pesticides, especially fungicides and herbicides, using the effect of a water-soluble β-CD polymer on the apparent pesticide’s lipophilicity [63]. Also, partial least square (PLS) modeling was used for the determination of the composition of solutions containing tryptophan methyl ester, phenylalanine, norephedrine, *N*,*N*’-bis-(α-methylbenzyl)sulfamide, sulfaguanidine or sulfamethoxazole using the spectral data of the corresponding CD host–guest complexes [64–66].

**Figure 1:**
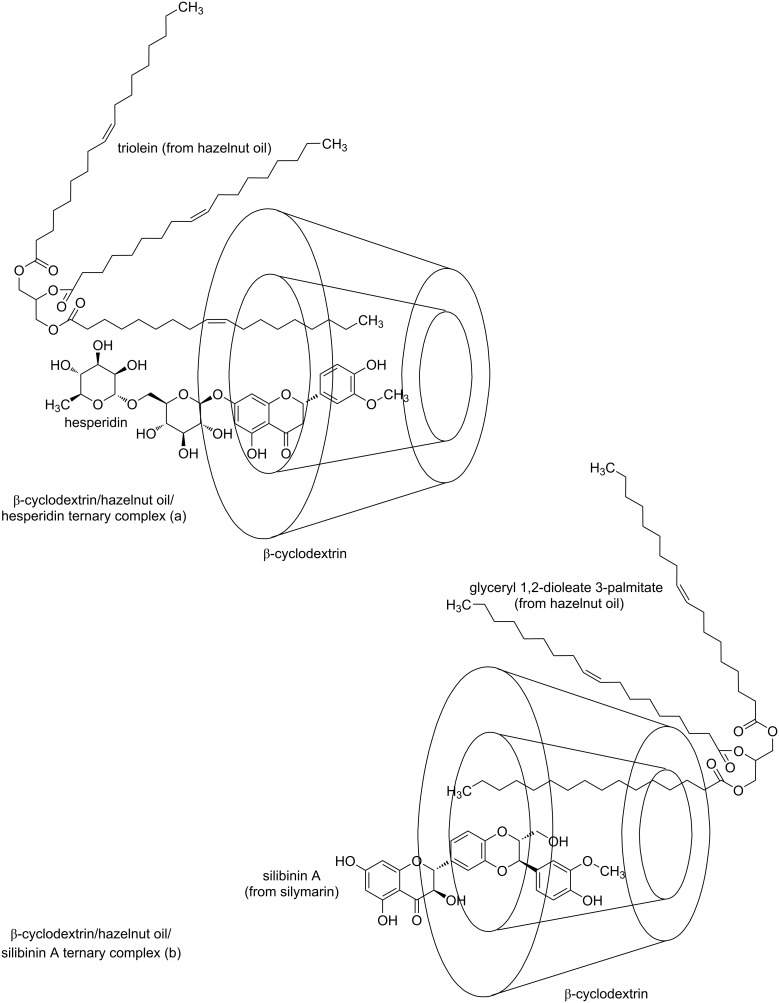
Hypothetical interactions between the β-cyclodextrin host and guest molecules (flavonoid glycoside/flavonolignan and a fatty acid triglyceride from the hazelnut oil), not explicitly proven in this study. (a) Both triolein from hazelnut oil and hesperidin interact with β-cyclodextrin from the secondary face; (b) glyceryl 1,2-oleate 3-palmitate from hazelnut oil interacts with the β-cyclodextrin from the primary face, while silibinin A, the main component of silymarin, interacts with β-cyclodextrin from the secondary face.

The goal of this study was the synthesis of β-CD/hazelnut (*Corylus avellana* L.) oil/flavonoid glycoside or flavonolignan ternary complexes (Figure 1) and the discrimination of these complexes by FTIR–PCA. These innovative ternary complexes were synthesized for the first time and can provide the on-site protection of hazelnut oil components against oxidative degradation, in combination with a protection/stabilization through CD nanoencapsulation. Moreover, the apparent water solubility, bioaccessibility, bioavailability, and controlled release of the guest bioactive compounds can also be enhanced by ternary complexation.

## Results and Discussion

### Synthesis and thermal analysis of the ternary complexes

The complexity of the starting materials, especially that of hazelnut oil, as well as the differences among their characteristics (hydrophobicity and water solubility) suggest the kneading method as the most appropriate one for obtaining β-CD/hazelnut (*Corylus avellana* L.) oil/flavonoid glycoside or flavonolignan ternary complexes. Kneading allows for higher recovery yields of complexes in comparison with the co-crystallization method because only small amounts of solvent are needed for preparation. On the other hand, similar methods such as spray-drying do not provide intimate contact between the three types of components for a sufficient period of time to reach the association–dissociation equilibrium [1,27,67]. In this study, the recovery yields were in the range of 51.5–85.3%, and significantly higher for the 3:1:1 complexes. Equimolar X1H, X1N, X1R and X1S ternary complexes were obtained with yields of 57.7 (± 8.8), 54.6 (± 1.9), 74.3 (± 1.8), and 64.7 (± 2.6)%, respectively. For the 3:1:1 ternary complexes (single samples) these yields were in the range of 74.5–85.3%. The difference in the yield can be explained by the level of hydration, as was determined by TG (see below). For the 1:1:1 complexes, the mass loss is half in comparison with the water content of β-CD (6.4–7.4% for complexes and 14% for β-CD hydrate). On the other hand, the mass loss of the 3:1:1 complexes is much higher (e.g., 11.8% for X3N complex). As a consequence, the 1:1:1 complexes lose relatively more hydration water than the corresponding 3:1:1 complexes. This can be explained by the high level of complexation for the 1:1:1 complexes. This aspect could be confirmed by thermal analysis, especially by DSC.

Both TG–DTG and DSC thermal analyses provide information about the molecular inclusion of guest molecules into the β-CD cavity. Unfortunately, these methods cannot differentiate between the encapsulated components and their entrapment efficiency. However, the goal of the study was the discrimination of such ternary complexes on the basis of FTIR. The evaluation of the encapsulation competitiveness of such multicomponent mixtures is very challenging (highly hydrophobic FA triglycerides, mono- and diglycerides, free FAs, as well as more hydrophilic flavonoid glycoside, namely hesperidin, naringin and rutin, or flavonolignan – silibinins). According to TG-DTG and DSC analyses, the ternary complexes are highly stable up to 200 °C. The TG and DTG plots were similar for ternary complexes at a 1:1:1 molar ratio, in comparison with the β-CD hydrate at temperatures up to ≈200 °C. The only significant difference was observed for the mass loss corresponding to water/moisture release up to ≈110 °C, with values of 6.37–7.38% and 9.45% for β-CD hydrate, respectively. A lower mass loss was observed for β-CD hydrate in comparison with the water content provided by the manufacturer (maximum 14% by oven drying). This could be due to the TG protocol, which assumes the pre-equilibration of the microbalance prior to analysis. Consequently, loss of surface water could have taken place before the start of the analysis. However, the difference of 2–3% for the ternary complexes at 1:1:1 molar ratios can be explained by a partial replacement of water molecules during the molecular encapsulation of the FA triglyceride and flavonoid guest molecules. On the other hand, the mass loss for the 3:1:1 ternary complexes was similar to the one observed of β-CD hydrate or even higher (see Supporting Information File 1, Figures S1–S4 and Tables S1 and S2). This means that a significant amount of β-CD is not involved in the formation of complexes and remains as β-CD hydrate in the mixture. These observations are in agreement with other studies on the complexation of vegetable (common bean lipids) and fish oil (common barbel, Pontic shad, European wels catfish, common bleak) by CDs [11,17]. Moreover, this TG behavior does not depend on the method of synthesis (kneading or co-crystallization) or the method of water determination (TG as mass loss or Karl Fischer water titration, KFT) [6,68]. It was observed that the difference between the water content or TG mass loss up to ≈110 °C is lower for binary complexes of CD/flavonoids in comparison with CD/fish oil (Atlantic salmon or European anchovy) [12,14,43]. The TG results are in agreement with the DSC data, where the calorimetric effect corresponding to water/moisture release is lower for the ternary complexes (378 J/g for X1N and 432 J/g for β-CD hydrate, Supporting Information File 1, Figure S5 and Table S3). There are two aspects that can be observed in the DSC but not in the TG–DTG analyses. The first aspect is the presence of two types of water molecules in the ternary complexes. They appear at two specific DSC peak temperatures of 44.5 °C for surface water and 82.0 °C for the stronger retained water molecules. While the surface water-related temperature is quite similar to β-CD hydrate, the stronger retained water has a higher DSC peak temperature value for β-CD (94.7 °C). This observation confirms the partial replacement of strongly retained water molecules during the complexation process. The second observation on DSC results is related to the absence of an endothermal–exothermal calorimetric peak in the case of the X1N ternary complex. This peak appears at 218.9 °C for β-CD hydrate and means that the complex obtained by kneading has an amorphous structure, in comparison with crystalline β-CD hydrate. The calorimetric peak observed for β-CD hydrate at this temperature is due to the transition of anhydrous β-CD (after water release) from the crystalline to the amorphous state [6]. Finally, TG analysis indicates a mass loss of 1.4–4.0% in the temperature range of 110–275 °C for the 1:1:1 ternary complexes and only 1.25% for the 3:1:1 complexes, whereas almost no mass loss was observed for β-CD hydrate (0.05%). The degradation of β-CD appears above 275 °C, with a maximum degradation rate at 299.4–326.0 °C as determined by DTG (the highest for β-CD) and at ≈322 °C by DSC. The degradation of the encapsulated hazelnut oil – of the triglyceride components – appears at a higher temperature of 394–407 °C (DTG and DSC).

### Fourier-transform infrared spectroscopy (FTIR) of ternary complexes

**Figure 2:**
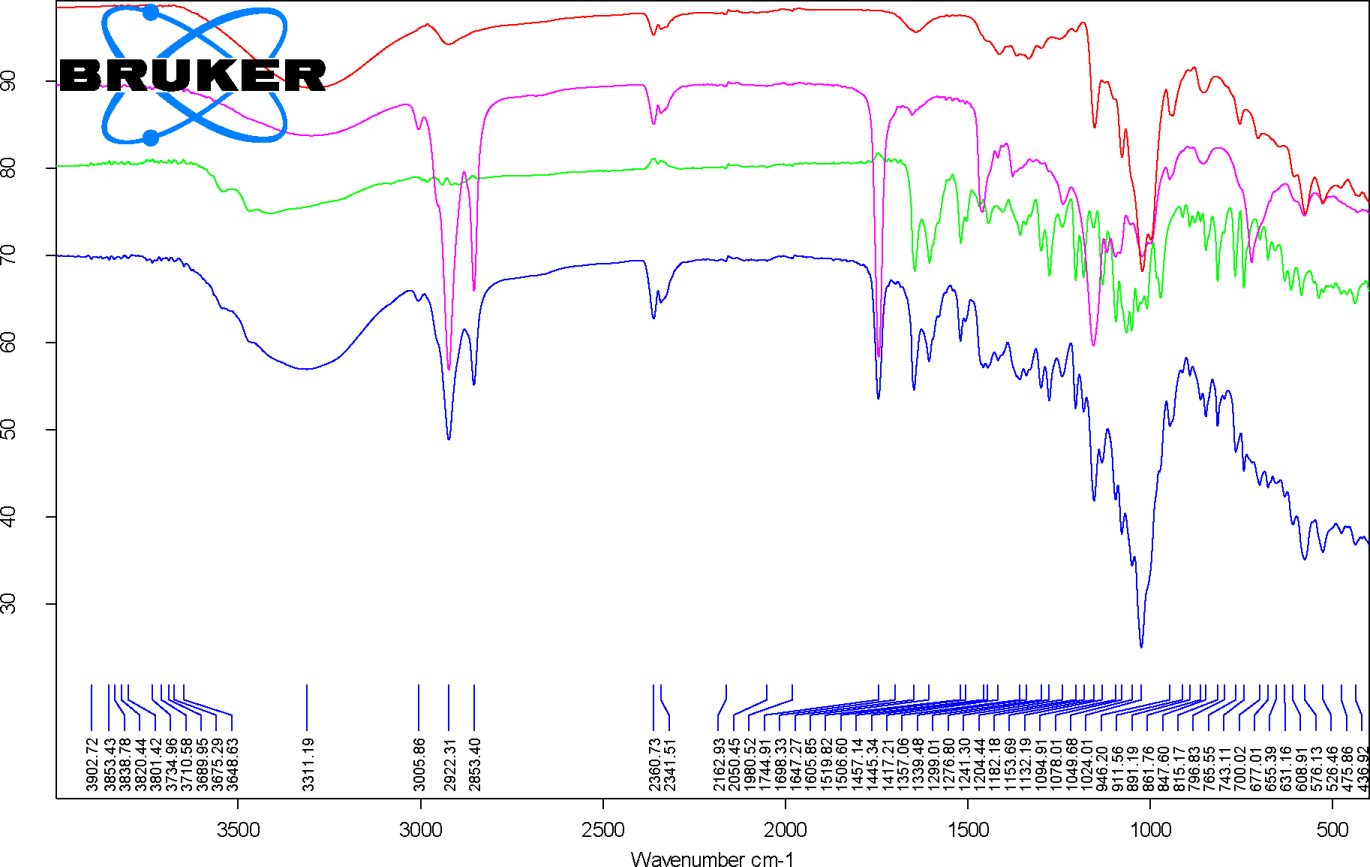
Superposition of the FTIR spectra for the β-cyclodextrin/*Corylus avellana* oil/hesperidin ternary complex at a 1:1:1 molar ratio (blue), β-cyclodextrin hydrate (red), *C. avellana* oil (pink), and hesperidin (green).

**Figure 3:**
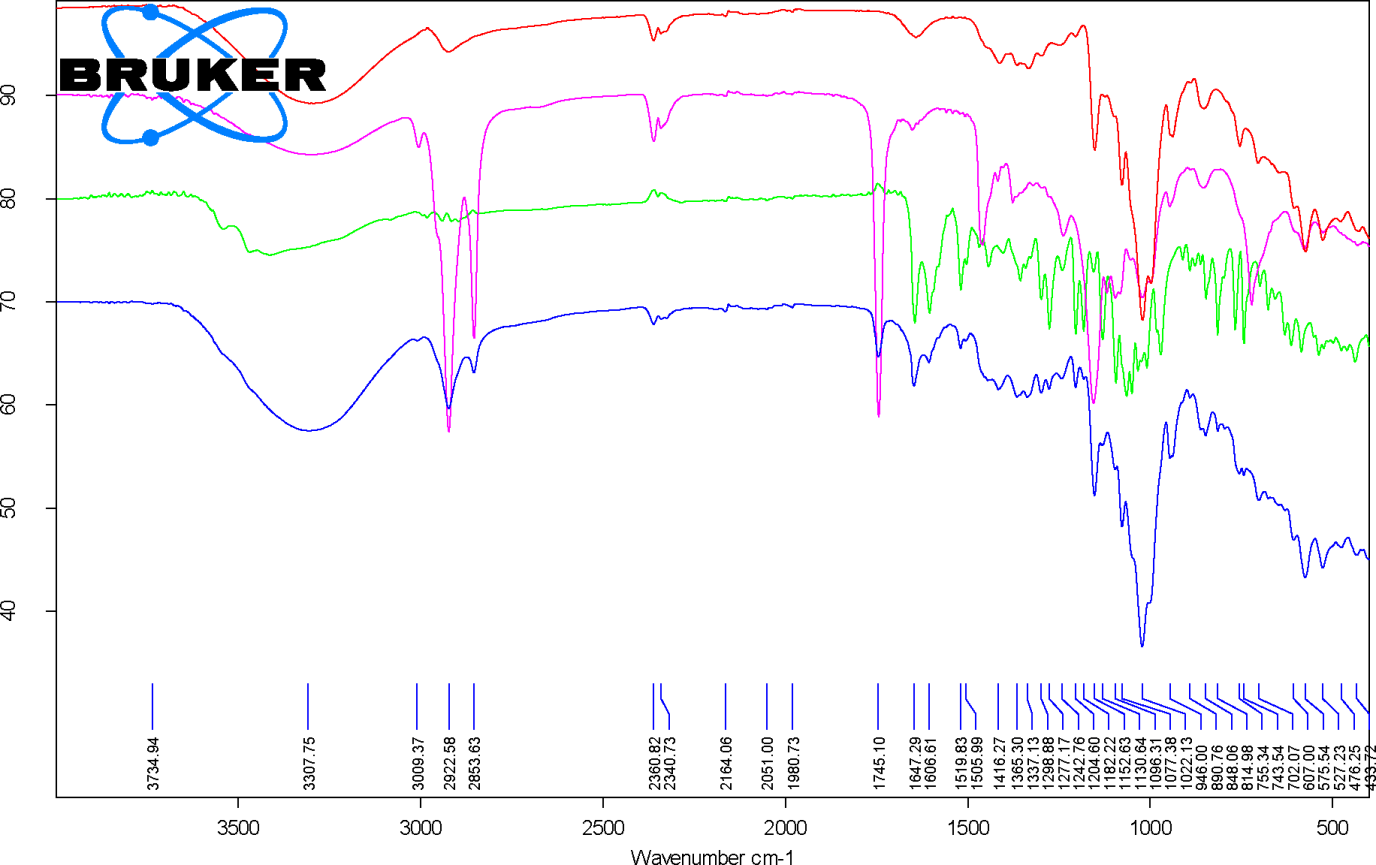
Superposition of the FTIR spectra for the β-cyclodextrin/*Corylus avellana* oil/hesperidin ternary complex at a 3:1:1 molar ratio (blue), β-cyclodextrin hydrate (red), *C. avellana* oil (pink), and hesperidin (green).

FTIR is a fast method that allows the evaluation of the presence of a compound in a complex through specific absorption bands. β-CD consists of seven 1→4-linked α-ᴅ-glucopyranose units forming a macrocycle. As a consequence, the FTIR specific bands especially appear for OH, CC and CH/CH_2_ bonds and groups. However, CD specific bands also appear for CH groups in the CD ring and α-type glycosidic bonds. Thus, a broad FTIR band corresponding to the stretching vibration of the O–H bonds in β-CD and hydration water molecules appears at ≈3301 cm^−1^. A weak band for the asymmetric stretching vibrations of the C–H groups appears at 2924.8 (± 1.4) cm^−1^, while the bending vibrations (in-plane, asymmetric, and symmetric) of the OH and CH groups appear as weak bands in the range of 1205–1643 cm^−1^. The stretching vibrations of the C–O and C–C groups in the glucoside moieties appear as medium-strong bands in the range of 998–1152 cm^−1^. A specific band for CD appears at 939.2 (± 1.8) cm^−1^ and is assigned to the stretching vibrations of the C–H groups from the β-CD ring. Also, the band at 852.9 (± 0.8) cm^−1^ is attributable to the bending vibrations of the C–C–H groups related to the α-type glycosidic bonds in the CD. Other bands appear at wavenumbers lower than 800 cm^−1^ and were tentatively assigned to the bending vibrations of the CH and OCC groups (574–754 cm^−1^), as well as to the stretching vibrations of the CC bonds at 526.3 (± 1.3) cm^−1^ [69–70]. Relevant data from the FTIR analysis of β-CD is presented in Figure 2 and Figure 3 and in Supporting Information File 1 (Figures S6–S11 and Table S4).

Vegetable oils and animal fat especially contain FA triglycerides, but mono-, diglycerides and free FAs also exist. As a consequence, the broad band corresponding to the stretching vibrations of the O–H groups is attributable to free fatty acids, monoglycerides, diglycerides and water. In the hazelnut samples, this band was observed at 3287.8 (± 10) cm^−1^. In this study, very useful was the weak band at 3005 (± 0.2) cm^−1^, which corresponds to the symmetric stretching vibrations of the =CH groups from the mono- and polyunsaturated FA moieties (especially oleic acid, but also palmitoleic and linoleic acids). The asymmetric and symmetric stretching vibrations of the CH groups provide strong bands at 2952.5 (± 0.3), 2922.5 (± 0), and 2853.2 (± 0) cm^−1^ due to the high number of CH_2_ and CH_3_ groups in the triglyceride structures. Another important and characteristic FTIR band for glycerides is that corresponding to the stretching vibrations of the ester C=O groups that appears as very strong band at 1744 (± 0) cm^−1^ for hazelnut oil. The stretching vibration of the *cis-*RHC=CHR’ group is observed as a weak band at 1652.7 (± 0.3) cm^−1^. Medium and strong bands are those related to the bending vibrations of the CH_2_ and CH_3_ groups at 1458.7 (± 0.2) cm^−1^, bending vibrations of the CH_2_ groups at 1236.8 (± 1.3) and 1158.1 (± 2.3) cm^−1^, the stretching vibrations of the C–O groups at 1027.9 (± 5.7) cm^−1^, as well as the out-of-plane bending vibrations in the C–H groups at 722 (± 0.1) cm^−1^. Degradation/isomerization of oil components (low level) can be observed at 956.7 (± 8.7) cm^−1^, where the band corresponding to the bending vibrations of the C=C groups in *trans-*RHC=CHR’ groups appears (sometimes at slightly higher values). Details of the FTIR analysis of hazelnut oil samples can be seen in Figure 2 and Figure 3 and in Supporting Information File 1 (Figures S6–S11 and Table S5) [71].

Hesperidin, naringin, and rutin are flavonoid glycosides derived from the corresponding flavanones hesperetin and naringenin and the flavonol quercetin, respectively. These compounds have a disaccharide moiety connected to the aglycones through an ether linkage with the hydroxy groups in the 7 and 3 positions (Figure 1a). On the other hand, silibinins (the main components of silymarin) are flavanonol derivatives, having a coniferyl alcohol moiety connected through the hydroxy groups in the 3’ and 4’ positions of the aglycone (Figure 1b). FTIR analysis of these flavonoids revealed stretching and bending vibrations corresponding to OH bonds (phenolic or alcoholic, glycosidic and OH groups from water molecules), CH bonds (especially from the CH_2_ and CH_3_ groups), bands corresponding to the aromatic CC bonds, and the carbonyl C=O bond. The most relevant FTIR band for these compounds is the asymmetric stretching vibration of the C=O bonds, ν^as^_C=O_, which appears around 1633–1651 cm^−1^. The lowest value for this band was observed for silymarin at 1634.1 (± 0.4) cm^−1^ and the highest one for rutin at 1651 (± 0.1) cm^−1^. For hesperidin and naringin this band appears at approximately the same value (≈1645 cm^−1^). The stretching vibrations of phenolic, glycosidic or water O–H bonds appear as broad bands in the range of 3263–3541 cm^−1^. Asymmetric and symmetric stretching vibrations of the C–H bonds in CH_3_ and CH_2_ groups appear at 2931–2941 cm^−1^. Similar FTIR bands also appear at 2982, 2907–2914, and 2876–2897 cm^−1^ in flavonoid glycosides. In the spectra of these compounds the bending vibrations of the aromatic CC groups appear at 1583–1604 cm^−1^ and ≈1518 cm^−1^, some of them being superimposed by the stretching vibrations of the C–C group in the ring C of aglycones. The stretching of a C–C group also appears in silymarin/silibinins at 1509.9 (± 0.6) cm^−1^, while this value is significantly lower for flavonoid glycosides (1502–1504 cm^−1^). Other bending vibrations were observed for CH bonds in the range of 1393–1468 cm^−1^, while the stretching vibrations for CC and CO bonds and the bending vibrations for HOC, OCH, an HCC groups were superimposed in the range of 1011–1364 cm^−1^. The stretching vibration of the O–C groups in all flavonoids appears at 968–995 cm^−1^. Finally, out-of-plane bending vibrations of CH groups and twisting bending vibrations of COH and HCCC groups appear in the range of 742–921 cm^−1^ [72–77]. All wavenumber values corresponding to the specific FTIR bands as well as the superimposed FTIR spectra of flavonoids with the other components of the ternary complexes are presented in Figure 2 and Figure 3 and in Supporting Information File 1 (Figures S6–S11 and Tables S6–S9).

The synthesized ternary complexes reveal the medium and strong FTIR bands of the above-mentioned host and guest components. However, FTIR bands that appear in specific regions where no interference occurs can also be relevant for the presence of individual compounds in the complex. This is the case for the weak band corresponding to the symmetric stretching vibrations of =CH groups from unsaturated glycerides in the hazelnut oil, which appear at 3006.5 (± 1), 3006.4 (± 0.6), 3006.3 (± 1.1), and 3006.6 (± 1.6) cm^−1^ for the X1H, X1N, X1R, and X1S ternary complexes at 1:1:1 molar ratios, respectively. These values are slightly higher by 1.1–3.1 cm^−1^ for all 3:1:1 ternary complexes (see Figure 2 and Figure 3 and Supporting Information File 1, Figures S6–S11 and Tables S6–S9). The strong bands corresponding to the asymmetric and symmetric stretching vibrations of the C–H bonds in the aliphatic CH_3_ and CH_2_ groups, as well as to the stretching vibrations of the ester C=O groups in triglycerides from hazelnut oil are clearly visible in all ternary complexes at 2922–2924, 2853–2854, and 1744–1745 cm^−1^, respectively. These values are very close to those corresponding to the starting hazelnut oil. Among other glyceride-related bands, those at 1453–1458 cm^−1^ originating from bending vibrations of the CH_2_ and CH_3_ groups, and 1236–1244 and 1152–1153 cm^−1^ from bending vibrations of the CH_2_ groups are also representative in the ternary complexes. They generally appear at lower values in the first case and at significantly higher values in the latter case in comparison with the starting hazelnut oil (see Supporting Information File 1, Figures S6–S11).

The most relevant flavonoid-related FTIR bands for the ternary complexes are those corresponding to the asymmetric stretching vibrations of the C=O groups. They occur in the range of 1637–1652 cm^−1^ for ternary complexes. The stretching vibrations of the C–C group in the ring C of the flavonoid glycosides or the bending vibrations of the aromatic CC groups occur in the range of 1598–1608 cm^−1^, but without specific variations in comparison with the starting compounds. The same is true for the band correlated to the in-plane bending vibrations of CH and OCH groups that appears at 1268–1299 cm^−1^. Also, the stretching vibrations of the C–C groups in the flavonoid glycosides or the stretching vibrations of the C–O groups in silymarin components (lower values) are observed in the same region. Another band that is present in all ternary complexes and is assigned to flavonoids is found at 807–821 cm^−1^, and corresponds to the out-of-plane bending vibrations of the C–H groups. This band appears at significantly lower values in rutin and rutin-related complexes.

β-CD was selected as the host for the formation of ternary complexes with the above-mentioned biologically active compounds and its content varies in complexes at 1:1:1 and 3:1:1 molar ratios. In the FTIR spectra of β-CD as a host, besides the wavenumbers corresponding to characteristic bands of β-CD, their intensities are relevant for the discrimination of the ternary complexes. However, many β-CD-related bands are weak or have at least medium intensities in the range of 1200–4000 cm^−1^. The most relevant bands for ternary complexes were the medium-strong intensity bands at 1152–1154 cm^−1^ (stretching vibrations of the C–O–C groups in the glucoside moieties), 1077–1080 cm^−1^ (stretching vibrations of the C–C groups), 1022–1026 cm^−1^ (stretching vibrations of the C–O groups), 944–947 cm^−1^ (stretching vibrations of the C–H groups from the β-CD ring), and two other medium intense bands at 574–576 and 522–529 cm^−1^, which were tentatively assigned as bending vibrations of the O–C–C groups and stretching vibrations of the C–C groups, respectively (see Figure 2 and Figure 3 and Supporting Information File 1, Figures S6–S11 and Tables S4, and S6–S9).

### Discrimination of ternary complexes by Fourier-transform infrared spectroscopy coupled with principal component analysis (FTIR–PCA)

Taking into account the differences between the wavenumbers and intensities of specific stretching and bending vibrations of β-CD hydrate, raw hazelnut oil, and flavonoids in the pure form and as ternary complexes, a multivariate statistical analysis technique was applied for the discrimination of these samples and identification of the important FTIR variables for such classifications. PCA is a widely used multivariate statistical analysis technique that can extract valuable information from a large dataset. It is the case of FTIR data (both wavenumbers and intensities), where were assigned 20, 17, 34, and 33 FTIR bands for β-CD hydrate, hazelnut oil, flavonoids, and ternary complexes, respectively (see Supporting Information File 1, Tables S4–S9). On the other hand, not all FTIR bands corresponding to the starting compounds can be observed and assigned for the ternary complexes. PCA works with a complete variable matrix. As a consequence, only the FTIR bands that were identified in both the starting materials and the ternary complexes were considered for PCA analysis (see Table 1 and Supporting Information File 1, Tables S10–S12). This matrix is transformed in order to obtain the maximum variance of the data. The new axes are denominated Factors or Principal Components (PCs). The translation coordinates will provide the scores plots that reveal the similarities/dissimilarities between cases (samples), while the representation of the rotation coordinates of the axes (direction cosines) will give information about the influence of variables to the classification of cases. Only few PCs will extract the useful information from the dataset. As a consequence, the large number of variables will be reduced to only 2–4 PCs that will explain the variance of the data.

#### Discrimination of flavonoid glycosides and flavonolignans

**Figure 4:**
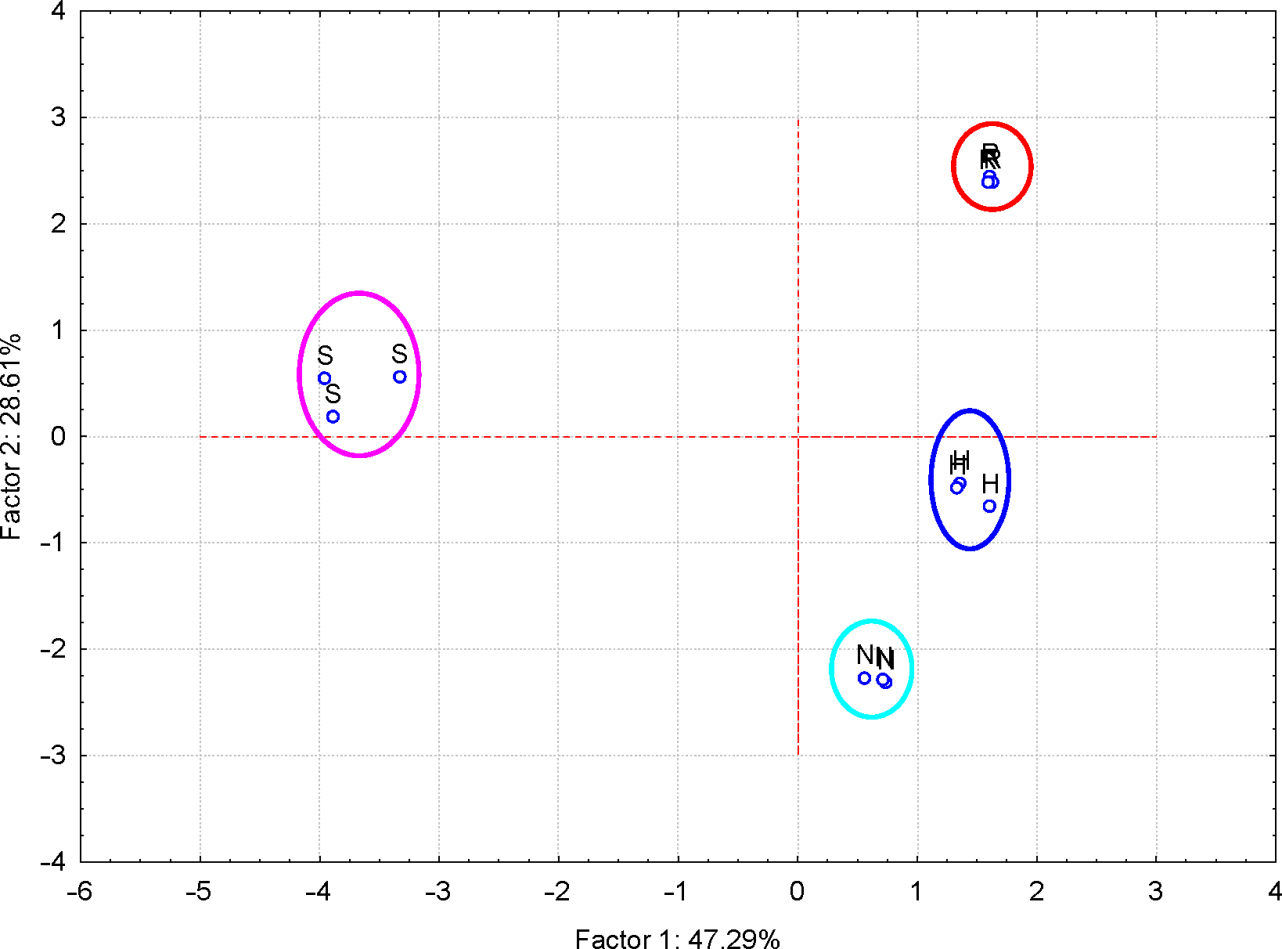
PC_2_ versus PC_1_ scores plot from the FTIR–PCA analysis of the flavonoid glycoside and flavonolignan antioxidants (codes: “H” – hesperidin, “N” – naringin, “R” – rutin and “S” – silymarin); only wavenumbers of the FTIR bands were used as input variables.

Twenty-two variables were considered for the discrimination of flavonoids (flavonoid glycosides – hesperidin, “H”, naringin, “N”, rutin, “R”, and flavonolignans – silymarin, “S”). They correspond to wavenumbers and intensities of the FTIR bands identified for all flavonoids (Supporting Information File 1, Table S10). The flavonoid samples were clearly grouped, according to the PC_2_ vs PC_1_ or PC_3_ vs PC_1_ scores plot (Supporting Information File 1, Figures S12 and S13). Better results were obtained when only wavenumbers were used as PCA variables (Figure 4). All flavonoid glycosides are classified in the positive region of the PC_1_, in comparison with flavonolignans (silymarin components). According to FTIR–PCA analysis, hesperidin, naringin, and rutin are more similar and all of them are dissimilar to silymarin. This classification is especially due to the bands corresponding to stretching vibrations of the C=O groups and bending vibrations for the CH groups for the positive region of PC_1_, as well as to the stretching vibrations of the CO and CC bonds for the negative part (Table 1 and Supporting Information File 1, Figures S14–S18 and Table S10). In this latter case, only the first three PCs explain 97.41% of the variance of the FTIR data, with the highest value for PC_1_ (47.29%; see the eigenvalues greater than 1 in Figure S19, Supporting Information File 1).

**Table 1:** Factor coordinates (principal components, PCs) of the variables, based on correlations from the FTIR–PCA analysis of the flavonoid glycoside and flavonolignan antioxidants; only wavenumbers (“v” – for stretching vibrations, “d” – for bending vibrations) of the FTIR bands were used as input variables.

	PC_1_	PC_2_	PC_3_

v(OH)	0.763	−0.616	−0.182
vas(CH)	−0.090	0.565	−0.780
vs(CH)	0.233	−0.781	−0.563
d(OH)/vas(C=O/C=C)	0.930	0.323	0.165
d(arC#C)	0.595	0.714	0.353
d1(CH2/3)	−0.350	0.026	−0.931
v1(CO)/d1(CO)	−0.416	0.797	−0.435
d1(CH)	0.986	−0.142	−0.061
v(CO)/v(CC)	0.937	0.128	−0.321
v(CO)/v(CC/CO)	−0.940	0.077	0.302
d4(CH)	−0.557	−0.739	0.049

#### Discrimination of ternary complexes and β-CD hydrate samples

**Figure 5:**
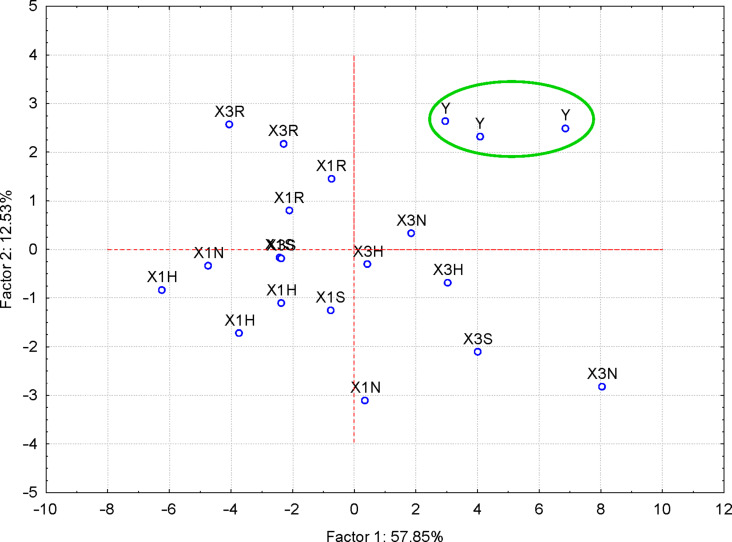
PC_2_ versus PC_1_ scores plot from the FTIR–PCA analysis of the β-CD/hazelnut oil/flavonoid ternary complexes (codes: “X1H/N/R/S” and “X3H/N/R/S” for the 1:1:1 and 3:1:1 ternary complexes with hesperidin/naringin/rutin/silymarin, respectively) and β-CD hydrate (code: “Y”); all wavenumbers and intensities of the FTIR bands were used as input variables.

In the same way, ternary complexes and native β-CD hydrate samples were classified according to specific FTIR wavenumbers and intensities of the bands identified in all samples. β-CD hydrate samples were classified in the top-right region of the PC_2_ vs PC_1_ scores plot (codes “Y”), in comparison with the ternary complexes in the center-left and bottom of the plot. Moreover, such grouping can also be observed for some ternary complexes types (e.g., “X1H” in the left and “X3R” in the top-left of the plot, Figure 5). Few FTIR variables are responsible for the discrimination of ternary complexes and β-CD samples, especially those related to band intensities corresponding to bending vibrations of CH_2_ groups and stretching vibrations of various bonds including those from CCO, CCC, CO and COC systems (PCA results are not presented).

#### Discrimination of ternary complexes and flavonoids

**Figure 6:**
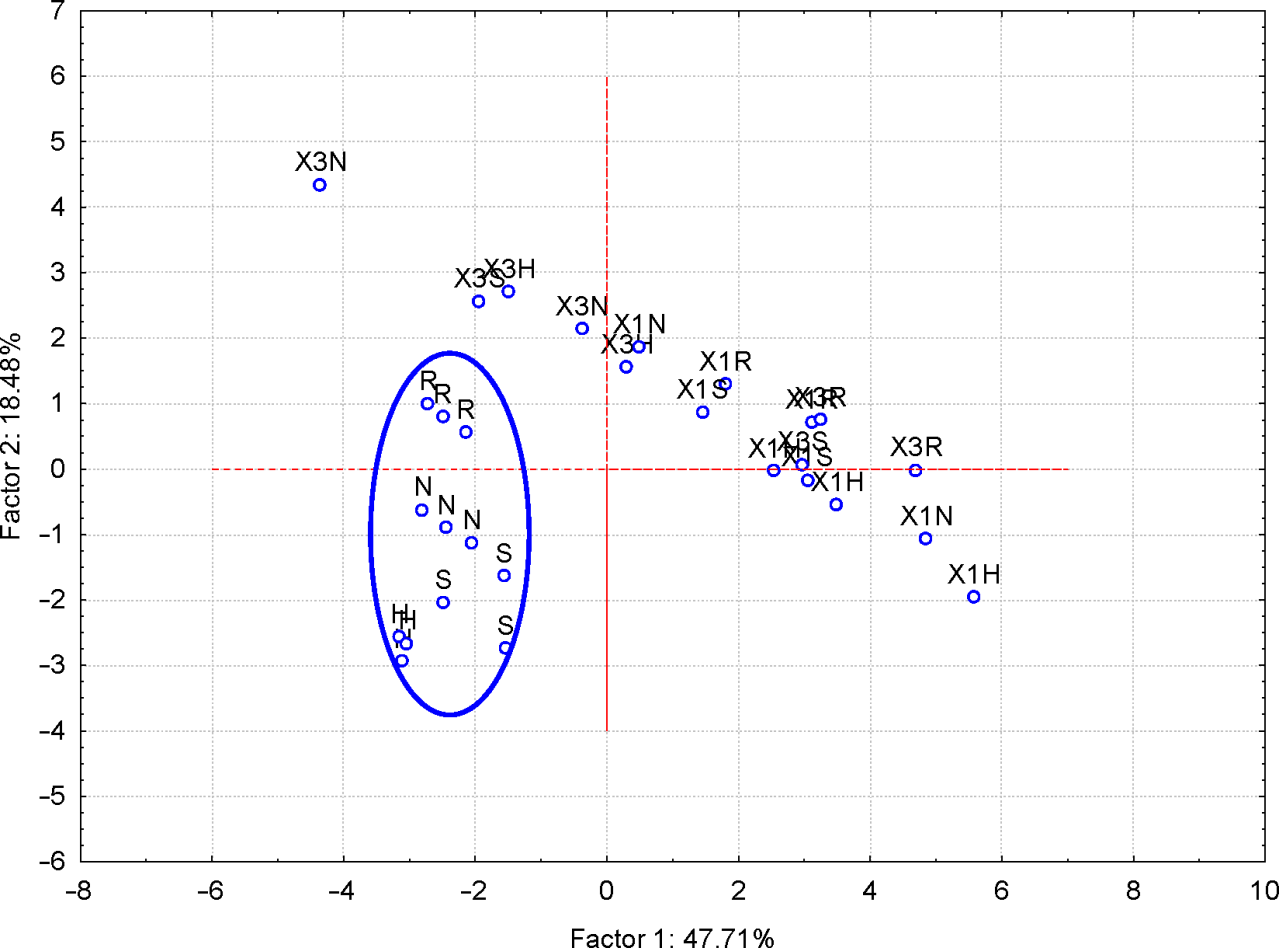
PC_2_ versus PC_1_ scores plot from the FTIR–PCA analysis of the β-CD/hazelnut oil/flavonoid ternary complexes (codes: “X1H/N/R/S” and “X3H/N/R/S” for the 1:1:1 and 3:1:1 ternary complexes with hesperidin/naringin/rutin/silymarin, respectively) and flavonoids (codes: “H” – hesperidin, “N” – naringin, “R” – rutin and “S” – silymarin); all wavenumbers and intensities of the FTIR bands were used as input variables.

**Figure 7:**
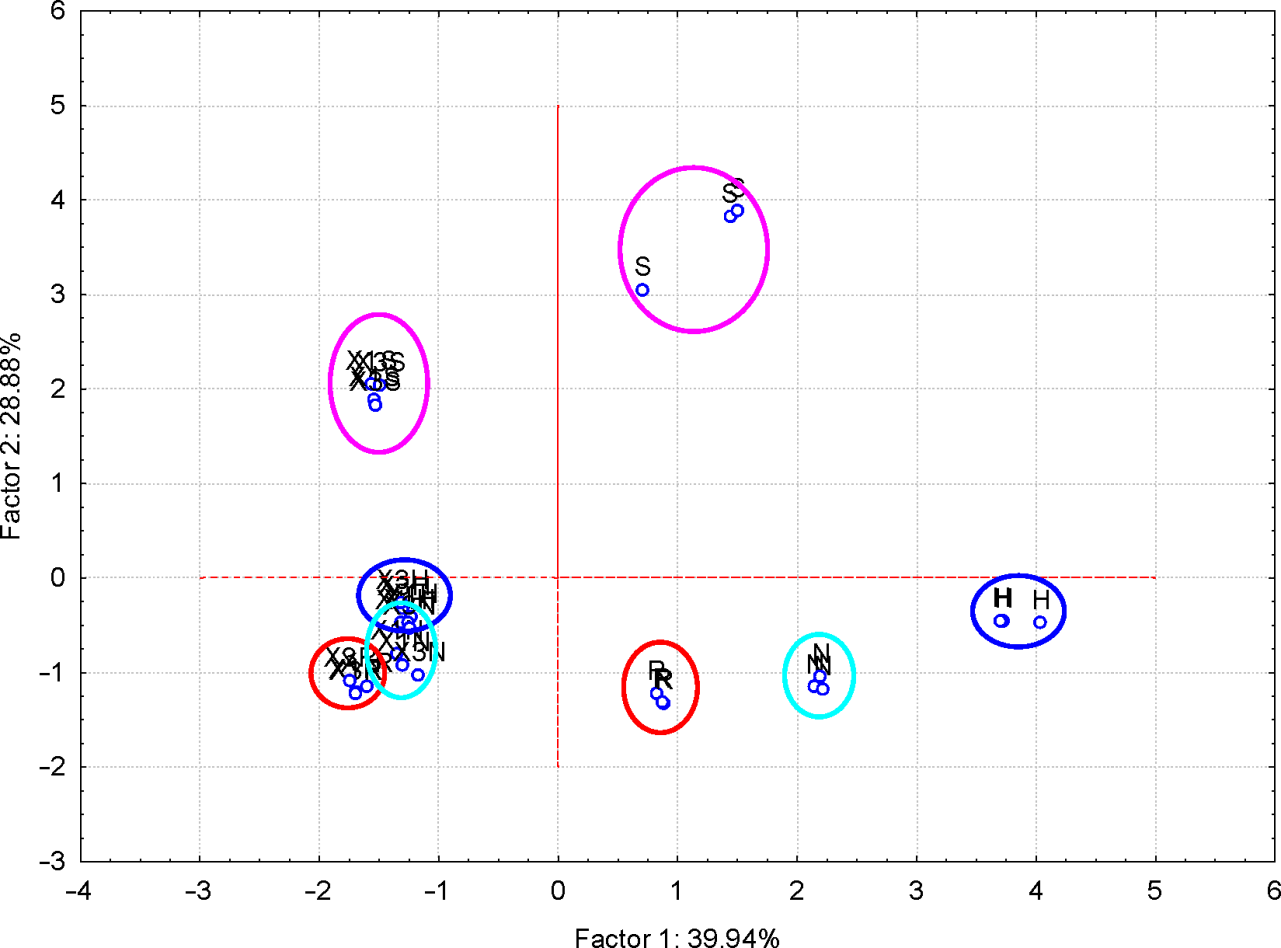
PC_2_ versus PC_1_ scores plot from the FTIR–PCA analysis of the β-CD/hazelnut oil/flavonoid ternary complexes (codes: “X1H/N/R/S” and “X3H/N/R/S” for the 1:1:1 and 3:1:1 ternary complexes with hesperidin/naringin/rutin/silymarin, respectively) and flavonoids (codes: “H” – hesperidin, “N” – naringin, “R” – rutin and “S” – silymarin); only wavenumbers of the FTIR bands were used as input variables.

**Figure 8:**
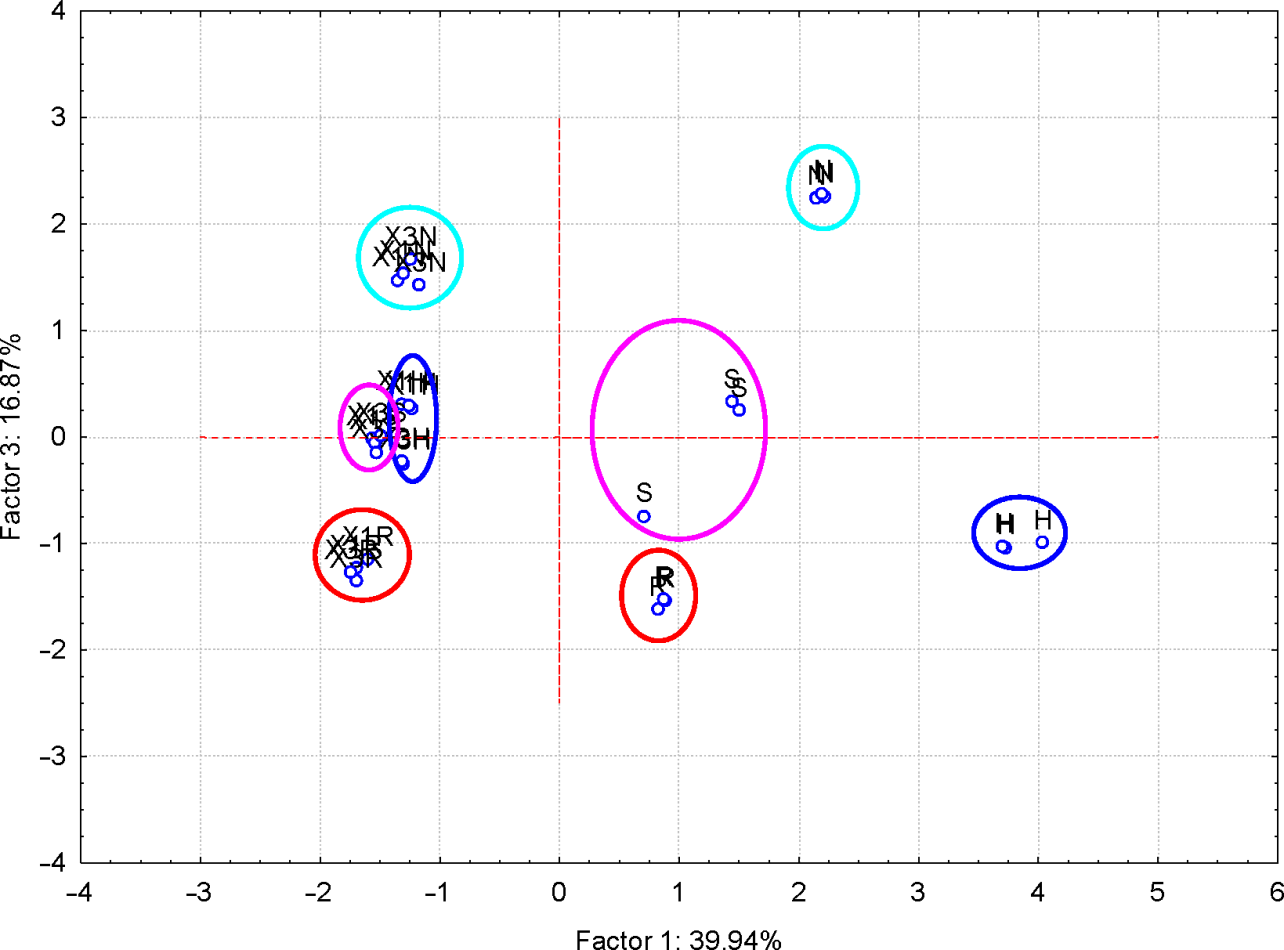
PC_3_ versus PC_1_ scores plot from the FTIR-PCA analysis of the β-CD/hazelnut oil/flavonoid ternary complexes (codes: “X1H/N/R/S” and “X3H/N/R/S” for the 1:1:1 and 3:1:1 ternary complexes with hesperidin/naringin/rutin/silymarin, respectively) and flavonoids (codes: “H” – hesperidin, “N” – naringin, “R” – rutin and “S” – silymarin); only wavenumbers of the FTIR bands were used as input variables.

**Figure 9:**
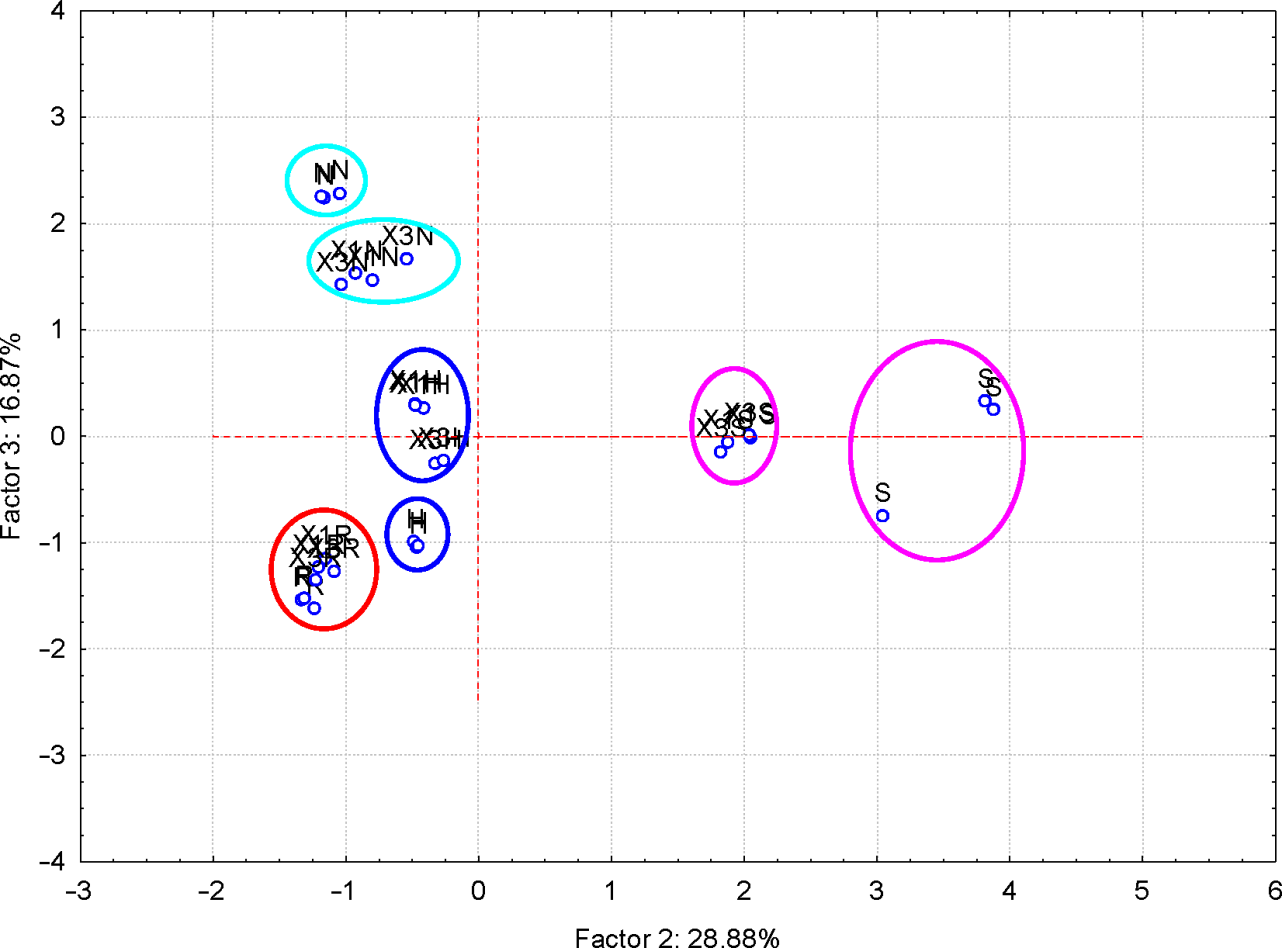
PC_3_ versus PC_2_ scores plot from the FTIR–PCA analysis of the β-CD/hazelnut oil/flavonoid ternary complexes (codes: “X1H/N/R/S” and “X3H/N/R/S” for the 1:1:1 and 3:1:1 ternary complexes with hesperidin/naringin/rutin/silymarin, respectively) and flavonoids (codes: “H” – hesperidin, “N” – naringin, “R” – rutin and “S” – silymarin); only wavenumbers of the FTIR bands were used as input variables.

**Figure 10:**
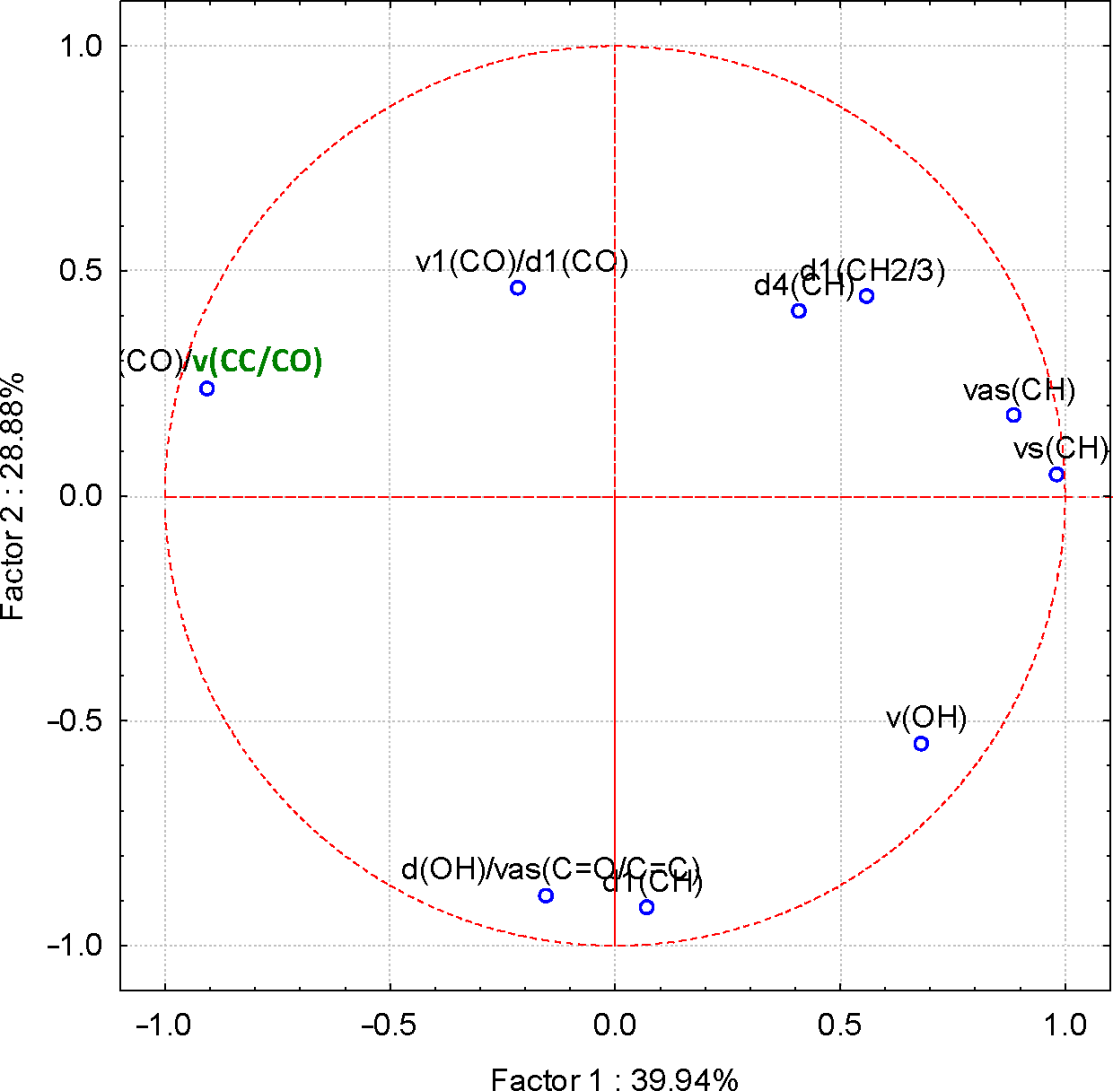
PC_2_ versus PC_1_ loadings plot from the FTIR–PCA analysis of the β-CD/hazelnut oil/flavonoid ternary complexes and flavonoids; only wavenumbers of the FTIR bands were used as input variables (see Table S12 in Supporting Information File 1 for codes).

**Figure 11:**
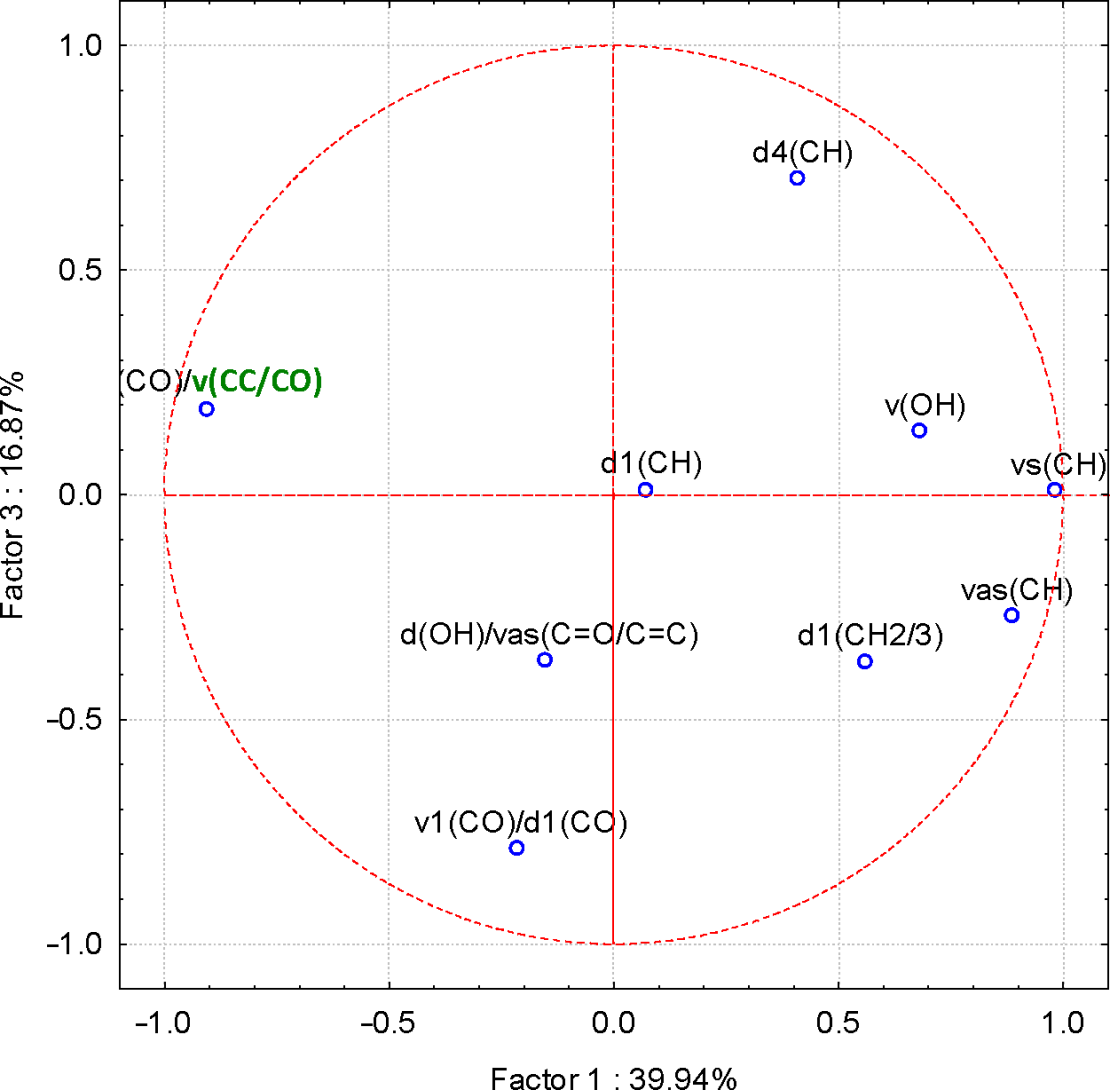
PC_3_ versus PC_1_ loadings plot from the FTIR–PCA analysis of the β-CD/hazelnut oil/flavonoid ternary complexes and flavonoids; only wavenumbers of the FTIR bands were used as input variables (see Table S12 in Supporting Information File 1 for codes).

**Figure 12:**
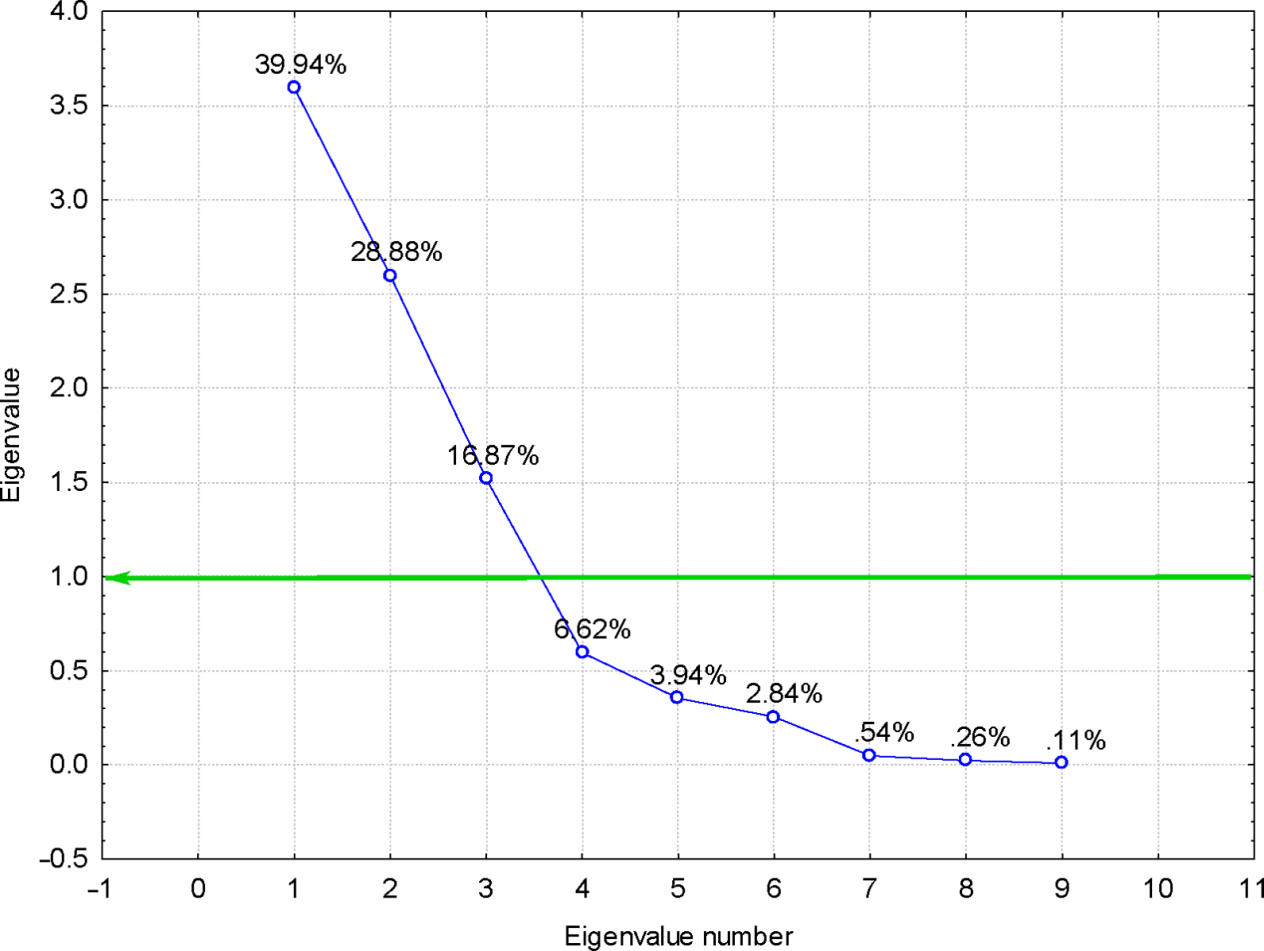
Eigenvalues of the correlation matrix from the FTIR–PCA analysis of the β-CD/hazelnut oil/flavonoid ternary complexes and flavonoids; only wavenumbers of the FTIR bands were used as input variables (see Table S12 in Supporting Information File 1 for codes); the first three PCs can be retained, which explain 85.69% from the variance of the data.

More interesting were the results obtained for the FTIR–PCA analysis of ternary complexes and flavonoids. A total of 18 FTIR variables (both wavenumbers and intensities, Supporting Information File 1, Tables S11 and S12) were identified in all ternary complexes and flavonoids. They were used as input variables for the discrimination of complexes and guest compounds. Also, the wavenumbers and intensities sets were used separately for the discrimination. Flavonoids were clearly classified in the left side of the PC_2_ vs PC_1_ scores plot (Figure 6). Wavenumbers of the bands corresponding to the stretching vibrations of the CO and CC bonds for the positive side, as well as the intensity of the band corresponding to the asymmetric stretching vibration of the CH bond for the negative side of the PC_1_ were the most important for this classification (see also Supporting Information File 1, Figure S20 for the PC_3_ vs PC_1_ scores plot, Figures S21and S22 for the corresponding loadings plots, and Table S11 for the influence of variables on the classification). Better results were obtained if only wavenumbers were used as input variables for the FTIR–PCA analysis of ternary complexes and the starting flavonoids. All flavonoids were grouped in the right side of the PC_2_ vs PC_1_ scores plot, with higher similarity for hesperidin, naringin, and rutin (Figure 7). On the other hand, all ternary complexes were located in the left side of this plot, also sub-classified according to the presence of specific flavonoids. In a similar manner, ternary complexes based on silymarin are dissimilar with the other complexes, which have a high level of similarity. These observations are also sustained by the other scores plots, all with very good classifications of the samples (Figure 8 and Figure 9). Responsible for these classifications are the variables corresponding to the FTIR bands related to symmetric and asymmetric stretching vibrations of the CH bonds (positive PC_1_), stretching vibrations of the CC and CO bonds (negative PC_1_), stretching and bending of C=O and OH/CH, respectively (negative PC_2_) (Figure 10 and Figure 11, Supporting Information File 1, Table S12). Only the first three PCs were used for obtaining these valuable discrimination results. They explain almost all variances of the FTIR data, as is presented in Figure 12 (85.69%).

## Conclusion

The β-CD/hazelnut oil/flavonoid ternary complexes are innovative materials synthesized for the first time, which combine the valuable properties of the specific components, the host – β-CD and the guests – the antioxidant and the essential FA glyceride compounds. β-CD encapsulation enhances the apparent water solubility of both hazelnut triglyceride components (e.g., triolein) and flavonoid glycosides/flavonolignans. They both have significantly lower water solubility and thus low bioaccessibility and bioavailability. They are enhanced by β-CD co-encapsulation. On the other hand, the encapsulated flavonoid molecule can act as on-site antioxidant and protect the labile hazelnut oil components that contain unsaturated FA moieties. The thermal/oxidative stability of ternary complexes is similar to β-CD hydrate, as was evaluated by TG and DSC. Moreover, the formation of the molecular inclusion complexes is supported by thermal analysis (partial replacing of the hydration water by biologically active molecules and disappearance of the DSC peak corresponding to crystalline–amorphous transition). In the present study, an appropriate synthesis method for ternary complexes (from the applicative point of view) was used. Also, a very fast, cheap and nondestructive technique, namely FTIR–PCA, was used for discrimination between ternary complexes (by the antioxidant used or by the molar ratio) and the starting components. β-CD/hazelnut oil/flavonoid ternary complexes at a 3:1:1 ratio had spectroscopic and thermal behavior more close to the native β-CD hydrate, in comparison with the 1:1:1 complexes. This observation indicates that not all FA moieties interact with the β-CD host molecules. This was the reason to use such non-equimolar ratios. If a theoretical 3:1 interaction can be considered, the formation of such β-CD/triglyceride supramolecular system in practice is limited by the steric hindrance. On the other hand, ternary complexes and flavonoids were very well classified and discriminated by FTIR–PCA, especially through the type of antioxidant used. However, further synthesis methods and analyses (slow co-crystallization, single-crystal X-ray diffraction, ^1^H and ^13^C nuclear magnetic resonance analyses) are needed for the elucidation of the interactions in such complex supramolecular systems.

## Experimental

### Vegetable samples and chemicals

Hazelnut (*Corylus avellana* L.) oil was obtained from nut kernel by Soxhlet extraction. Wild hazelnuts were collected from the Apuseni Mountains (Transylvania, Romania, 46°22’46” N and 23°16’47” E) between September and October 2018 and were kept at room temperature, in the dark, and dry atmosphere for six months. Then, the kernels were manually separated, finely ground, and subjected to Soxhlet extraction using a 250 mL equipment. One hundred of hazelnut kernels were extracted five times with 300 mL of anhydrous petroleum ether (ACS reagent, 40–60 °C boiling range, Sigma-Aldrich, St. Louis, MO, USA). The extract was distilled and evaporated to dryness until no petroleum ether remained. The oil separation yield was ≈50%. The hazelnut oil was kept at −20 °C until further analyses and β-CD complexation.

β-CD hydrate, Kleptose^®^, was kindly donated by Roquette Frères S.A. (Lestrem, France) and had a purity of >98%, a water content of 14.0%, and maximum 0.5% α-CD and γ-CD. Flavonoid glycosides and flavonolignans used in the complexation process were hesperidin (code “H”, C_28_H_34_O_15_, M = 610.56 g/mol, purity ≥80%, other flavonoid glycosides as impurities), naringin hydrate (code “N”, C_27_H_32_O_14_·2H_2_O, M = 580.50 g/mol, purity ≥95%), rutin hydrate (code “R”, C_27_H_30_O_16_·*x*H_2_O, M = 610.52 g/mol, purity ≥94%), and silymarin (code “S”, C_25_H_22_O_10_, M = 482.44 g/mol, ≈70% silibinin A, other flavonolignans as impurities) and were purchased from Sigma-Aldrich, St. Louis, MO, USA. Ethanol used for complex synthesis was of 96% concentration (v/v) and was purchased from ChimReactiv (Bucharest, Romania). The analysis of the FA profile of the hazelnut oil required the derivatization (transesterification) of the FA glycerides to the corresponding FA methyl esters (FAMEs) [11,13]. The derivatization involved methanol–boron trifluoride (20% BF_3_), hexane (GC grade) and anhydrous sodium sulfate, all purchased from Merck & Co., Inc., Rahway, NJ, USA. Sodium chloride (reagent grade) used for the separation of FAMEs was purchased from Reactivul (Bucharest, Romania). The identification of the FAME components of the hazelnut oil involved FAME37 standard mixture, as well as C_8_–C_20_ linear alkane standard mixture for the determination of the specific retention index (RI) of compounds (both purchased from Sigma-Aldrich, St. Louis, MO, USA). Finally, 2-propanol (ACS reagent, Reag. Ph. Eur.) used for FTIR cleaning was obtained from Merck & Co., Inc., Rahway, NJ, USA.

### Gas chromatography–mass spectrometry (GC–MS)

The FA profile of the hazelnut oil was determined by GC–MS, after derivatization to FAMEs. Derivatization was performed by quantitative transesterification in a 100 mL one-necked flask equipped with reflux condenser. 5 mL of BF_3_·MeOH 20% and ≈100 mg of hazelnut oil were used for derivatization. The mixture was refluxed for at least 30 min, until no oil remained. Then, 2 mL of hexane was added and the mixture refluxed for another 15 min for completing the transesterification. The organic layer was separated in the neck region by adding a sufficient amount of saturated sodium chloride solution. The organic layer was transferred into a GC vial with ≈0.5 g of anhydrous sodium sulfate and stored at 4 °C until GC–MS analysis. GC–MS analysis was performed on a GC Hewlett Packard 6890 Series equipment, coupled with a Hewlett Packard 5973 Mass Selective Detector. The following GC conditions were used: Zebron 5-MS column (30 m length, 0.25 mm i.d., 0.25 µm film thickness), temperature program of 50–300 °C (heating rate 6 °C/min), injector temperature 300 °C, detector temperature 300 °C, carrier gas He (99.9999% purity), injected sample volume 2 µL, delay time 4 min. The MS conditions were: energy source EI 70 eV, temperature 150 °C, scan range 50–300 amu, scan rate 1/s. RI values were determined using a C_8_–C_20_ alkane standard mixture and a RI vs RT correlation equation of RI = 672.792 + 73.268·RT − 3.287·RT^2^ + 0.148·RT^3^ − 0.00201·RT^4^ [16]. On the other hand, the identification of the main FAMEs from the derivatized hazelnut oil was performed by comparing the experimental RI values with those for the FAME standard mixture. Moreover, the experimental MS spectra were compared with those from the NIST/EPA/NIH Mass Spectral Library 2.0 (2011). Acquisition and handling of the GC–MS data were performed using the Enhanced MSD ChemStation D.02.00.275 (Agilent Technologies, Santa Clara, CA, USA), while the MS identification was performed with the NIST Mass Spectral Search Program for the NIST/EPA/NIH Mass Spectral Library 2.0 (Gaithersburg, MD, USA). Determinations were performed in duplicate and the main findings reveal a high oleic acid relative content (as methyl ester) of 69.91(± 4.14) % at a RI of 2096.4. The other important FAs, as methyl esters, were palmitoleic, palmitic, linoleic, elaidic/vaccenic, and stearic acids with concentrations of 0.13, 7.54, 15.51, 2.85 and 2.73%, respectively (a total of 98.68% identified FAMEs in the hazelnut oil).

#### Synthesis of ternary complexes by the kneading method

The synthesis of β-CD/hazelnut oil/flavonoid glycoside or flavonolignan ternary complexes was performed using the kneading method, which is the most appropriate for such type of complexes [13–14,50]. In this study, two β-CD:hazelnut oil:flavonoid molar ratios of 1:1:1 and 3:1:1 were used. Particularly, 1322 (± 5) or 3959 (± 10) mg of β-CD hydrate (for 1:1:1 and 3:1:1 molar ratios, respectively), 909 (± 5) mg hazelnut oil, 613 (± 3) mg hesperidin, 628 (± 5) mg naringin hydrate, 656 (± 5) mg rutin hydrate and 488 (± 1) mg silymarin were weighted, taking into account the water content and purity of compounds. The mean molar mass for the hazelnut oil of M = 900 g/mol was determined as triolein, according to GC–MS data and a purity of ≈97% [33,78]. The following ternary complexes were obtained: β-CD/hazelnut oil/hesperidin at 1:1:1 and 3:1:1 molar ratios (codes “X1H” and “X3H”), β-CD/hazelnut oil/naringin at 1:1:1 and 3:1:1 molar ratios (codes “X1N” and “X3N”), β-CD/hazelnut oil/rutin at 1:1:1 and 3:1:1 molar ratios (codes “X1R” and “X3R”) and β-CD/hazelnut oil/silymarin at 1:1:1 and 3:1:1 molar ratios (codes “X1S” and “X3S”). The amounts of β-CD, hazelnut oil, and flavonoid, corresponding to 1:1:1 or 3:1:1 were mixed in a preheated mortar at 60 °C. Then, 4 mL water and 1 mL ethanol for 1:1:1 complexes or 6 mL water and 1.5 mL ethanol for 3:1:1 complexes were added. The mixture was kneaded for at least 30 min, until a viscous paste is obtained. The mortar temperature decreases to the room temperature during kneading. The wet complex was dried until constant mass at room temperature in the dark. The dried complex was then grinded in the same mortar, recovered and weighted. The recovering yield was determined as the percent ratio of the recovered dried complex and the sum of starting compounds. The 1:1:1 ternary complexes were obtained as duplicate samples, while the 3:1:1 ternary complexes were obtained as unique samples.

#### Fourier-transform infrared spectroscopy (FTIR)

FTIR analysis of the ternary complexes and the starting compounds was performed using a Bruker Vertex 70 FTIR equipment (Bruker Optik GmbH, Ettlingen, Germany), equipped with an ATR (single-reflection Platinum diamond attenuated total reflectance) system. The following FTIR conditions were set up: acquisition range 4000–400 cm^−1^, resolution 4 cm^−1^, number of scans 128, sample mass 10–20 mg, spectrum range for the DLaTGS detector 12000–250 cm^−1^ and sensibility D* > 2108 cm·Hz^1/2^·W^−1^. OPUS ver. 7.2 software (Bruker Optik GmbH 2012, Ettlingen, Germany) was used for the acquisition and handling of the FTIR spectra. All determinations were performed as triplicates for the starting compounds and as duplicates for the ternary complexes.

#### Thermal analyses

The thermal and oxidative stability of complexes can be evaluated through thermal analyses. TG–DTG and DSC techniques were used for both the complexes and starting compounds. TG–DTG analysis was performed on a Netzsch TG 209F1 Libra equipment, while DSC analysis was conducted on a Netzsch 204 F1 Phoenix apparatus (both from Netzsch Group, Selb, Germany). The TG–DTG and DSC conditions were similar: temperature program of 25–500 °C, with a heating rate of 10 °C/min, nitrogen purge and protection flow of 40 mL/min, the data acquisition and handling by Netzsch Proteus-Thermal Analysis ver. 6.1 software (Netzsch Group, Selb, Germany). Only representative ternary complexes were evaluated by thermal analyses.

#### Statistical analysis and principal component analysis (PCA)

Means (± standard deviations, SD) of the values were obtained for the replicate determinations using Basic Statistics&Tables and One-way ANOVA modules in Statistica 7.1 software (StatSoft, Inc., Tulsa, OK, USA). PCA for the FTIR data was performed with the Principal Components & Classification Analysis module from the above-mentioned package. The discrimination between samples was based on the scores plot, while the importance of variables to the classification was based on the loadings plot in PCA analysis. Both FTIR wavenumber (WN) and intensity (I) of the specific bands identified in all analyzed samples were used as input data. PCA was performed with both FTIR variable types (both WN and I) or as separated variable types (only WN or only I). PCA analysis was based on correlations, a computed variance as SS/(N-1), with centered factor coordinates of the variables (or principal components, coded as “PC”). All significant PCA results are also presented in the Supporting Information File 1 (Figures S12–S23 and Tables S10–S12).

## Supporting Information

File 1 Thermal analysis, FTIR and FTIR–PCA data for ternary complexes.
