# Supplementary material for: Discrimination of β-cyclodextrin/hazelnut (Corylus avellana L.) oil/flavonoid glycoside and flavonolignan ternary complexes by Fourier-transform infrared spectroscopy coupled with principal component analysis
Source: Beilstein J Org Chem. 2023 Mar 28;19:380–98. doi: 10.3762/bjoc.19.30 (PMC10071518; doi:10.3762/bjoc.19.30)
Supplement: File 1 — Thermal analysis, FTIR and FTIR–PCA data for ternary complexes. [file Beilstein_J_Org_Chem-19-380-s001.pdf]

BEILSTEIN JOURNAL OF ORGANIC CHEMISTRY

Supporting Information

for

Discrimination of β-cyclodextrin/hazelnut (*Corylus avellana* L.) oil/flavonoid glycoside and flavonolignan ternary complexes by Fourier-transform infrared spectroscopy coupled with principal component analysis

Nicoleta G. Hădărugă, Gabriela Popescu, Dina Gligor (Pane), Cristina L. Mitroi, Sorin M. Stanciu and Daniel Ioan Hădărugă

Beilstein J. Org. Chem. **2023,** *19,* 380–398. doi:10.3762/bjoc.19.30

Thermal analysis, FTIR and FTIR–PCA data for ternary complexes

License and Terms: This is a supporting information file under the terms of the Creative Commons Attribution License (https://creativecommons.org/licenses/by/4.0). Please note that the reuse, redistribution and reproduction in particular requires that the author(s) and source are credited and that individual graphics may be subject to special legal provisions.
The license is subject to the *Beilstein Journal of Organic Chemistry* terms and conditions: (https://www.beilstein-journals.org/bjoc/terms)

1. Thermal analyses (TG–DTG and DSC) of ternary complexes

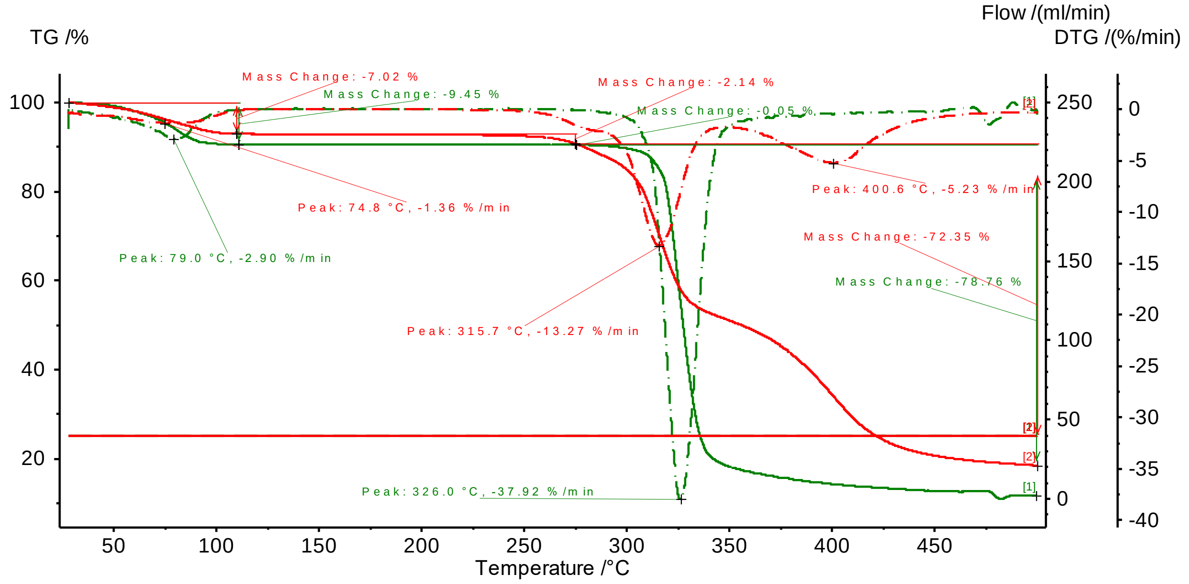

Figure S1: Superimposed TG–DTG thermograms for β-cyclodextrin/hazelnut oil/hesperidin ternary complex at a 1:1:1 molar ratio (code X1H, red) and β-cyclodextrin hydrate (green).

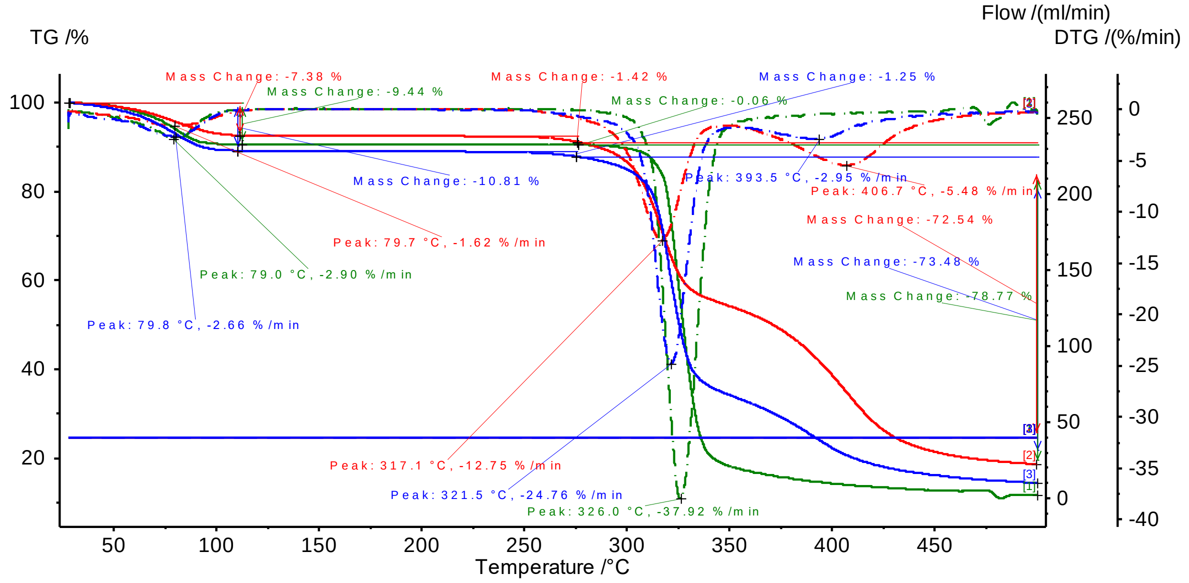

Figure S2: Superimposed TG–DTG thermograms for β-cyclodextrin/hazelnut oil/naringin ternary complex at 1:1:1 (code X1N, red) and 3:1:1 molar ratios (code X3N, blue), in comparison with the β-cyclodextrin hydrate (green).

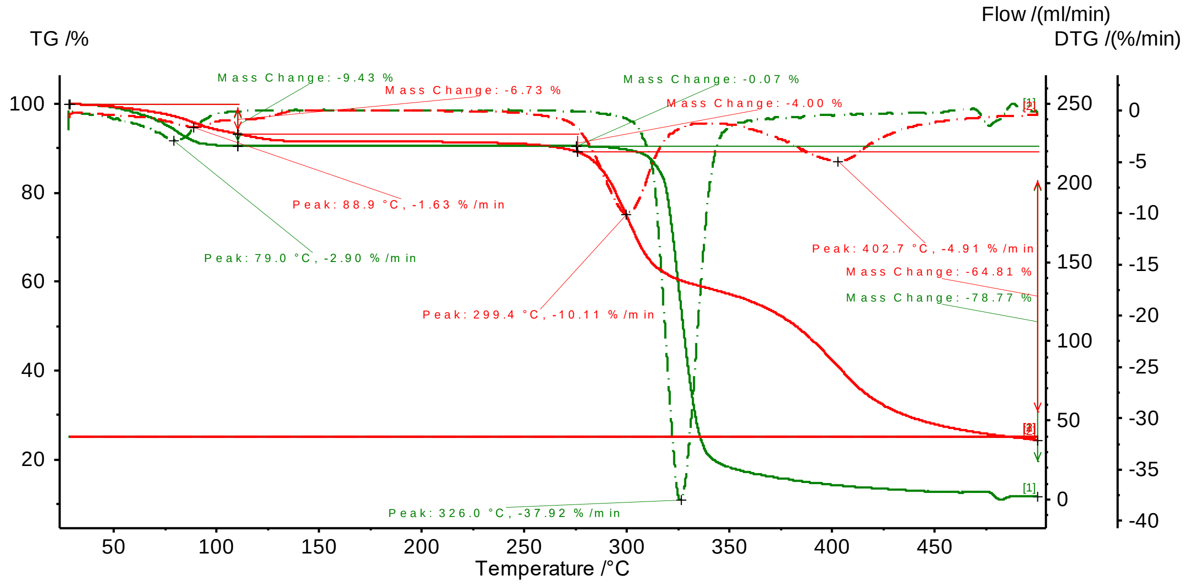

Figure S3: Superimposed TG–DTG thermograms for β-cyclodextrin/hazelnut oil/rutin ternary complex at a 1:1:1 molar ratio (code X1R, red) and β-cyclodextrin hydrate (green).

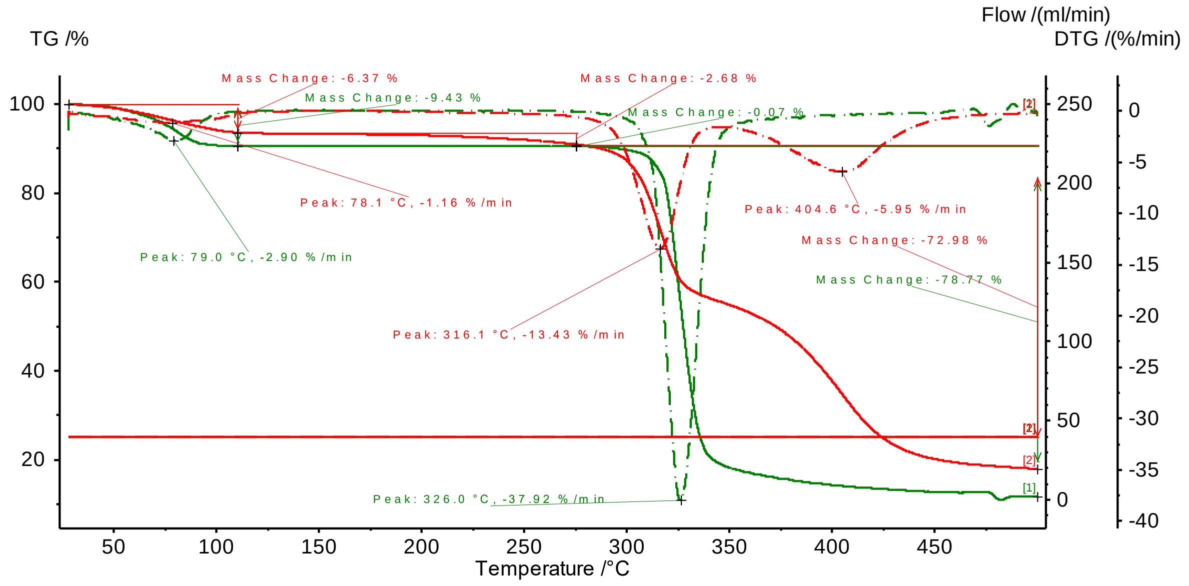

Figure S4: Superimposed TG–DTG thermograms for β-cyclodextrin/hazelnut oil/silymarin ternary complex at a 1:1:1 molar ratio (code X1S, red) and β-cyclodextrin hydrate (green).

Table S1: TG results (mass loss, %, for specific temperature ranges) for β-cyclodextrin hydrate (β-CD) and the β-cyclodextrin/hazelnut oil/flavonoid glycoside or flavonolignan ternary complexes at 1:1:1 or 3:1:1 molar ratios (codes X1H, X1N, X3N, X1R and X1S).

No	Code	Mass loss$_{(<110\ °C)}$ (%)	Mass loss$_{(110–275\ °C)}$ (%)	Mass loss$_{(>275\ °C)}$ (%)
1	β-CD	9.45	0.05	78.76
2	X1H	7.02	2.14	72.35
3	X1N	7.38	1.42	72.54
4	X3N	10.81	1.25	73.48
5	X1R	6.73	4.00	64.81
6	X1S	6.37	2.68	72.98

Table S2: DTG results (peak temperatures for the maximum mass loss rate, °C, for specific temperature ranges) for β-cyclodextrin hydrate (β-CD) and the β-cyclodextrin/hazelnut oil/flavonoid glycoside or flavonolignan ternary complexes at 1:1:1 or 3:1:1 molar ratios (codes X1H, X1N, X3N, X1R and X1S).

No	Code	$T_{DTG1(<110\ °C)}$ (°C)	$T_{DTG2(275–350\ °C)}$ (°C)	$T_{DTG3(>350\ °C)}$ (°C)
1	β-CD	79.0	326.0	-
2	X1H	74.8	315.7	400.6
3	X1N	79.7	317.1	406.7
4	X3N	79.8	321.5	393.5
5	X1R	88.9	299.4	402.7
6	X1S	78.1	316.1	404.6

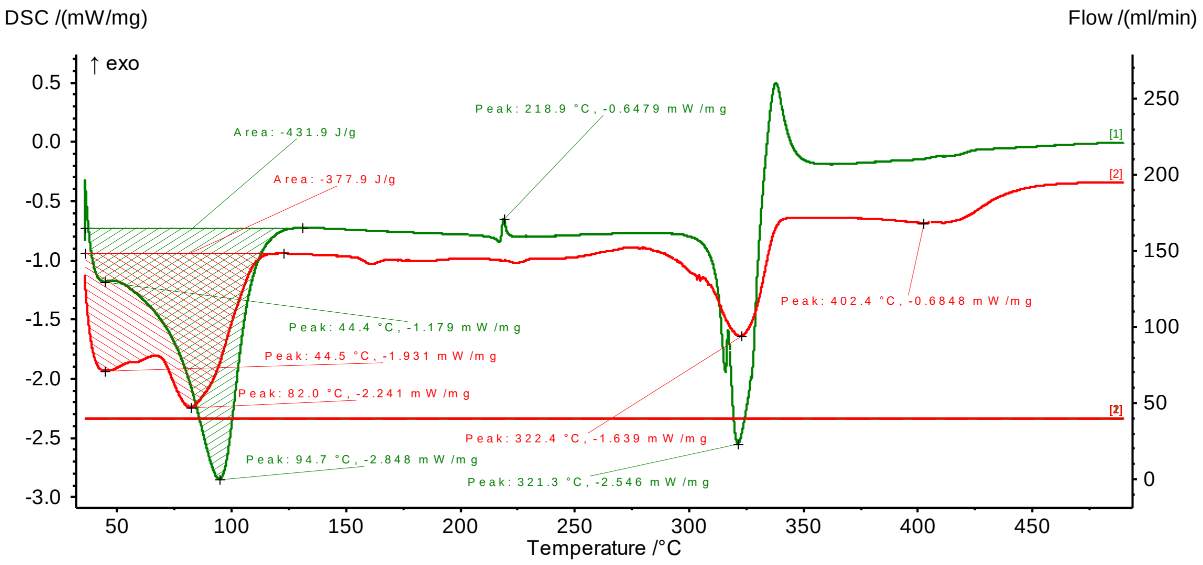

Figure S5: Superimposed DSC plots for the β-cyclodextrin/hazelnut oil/naringin ternary complex at 1:1:1 molar ratio (code X1N, red) and β-cyclodextrin hydrate (green).

Table S3: DSC results (peak area, J/g, and peak temperatures for the maximum rate of the calorimetric effect, °C, for specific temperature ranges) for β-cyclodextrin hydrate (β-CD) and the β-cyclodextrin/hazelnut oil/naringin ternary complex at a 1:1:1 molar ratio (code X1N).

No	Code	$Area_{DSC(<110°C)}$ J/g	$T_{DSC1(<110\ °C)}$ (°C)	$T_{DSC2(<110\ °C)}$ (°C)	$T_{DSC3(140–275\ °C)}$ (°C)	$T_{DSC4(275–350\ °C)}$ (°C)	$T_{DSC5(>350\ °C)}$ (°C)
1	β-CD	431.9	44.4	94.7	218.9	321.3	–
2	X1N	377.9	44.5	82.0	–	322.4	402.4

2. Fourier transform infrared spectroscopy (FTIR) of ternary complexes

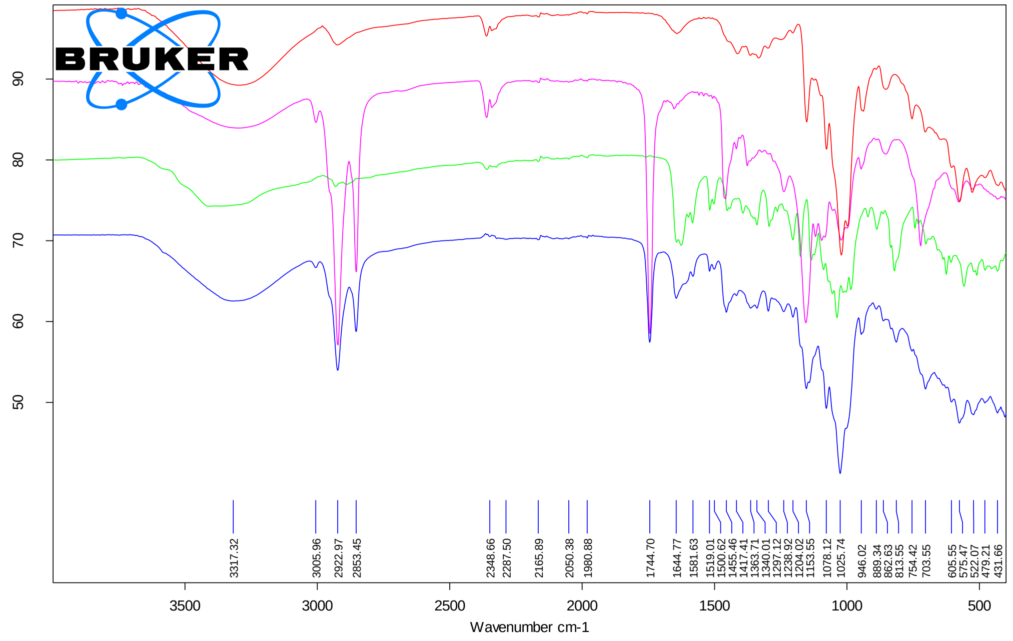

Figure S6: Superposition of the FTIR spectra for the β-cyclodextrin/*Corylus avellana* oil/naringin ternary complex at a 1:1:1 molar ratio (blue), β-cyclodextrin hydrate (red), *C. avellana* oil (pink), and naringin (green).

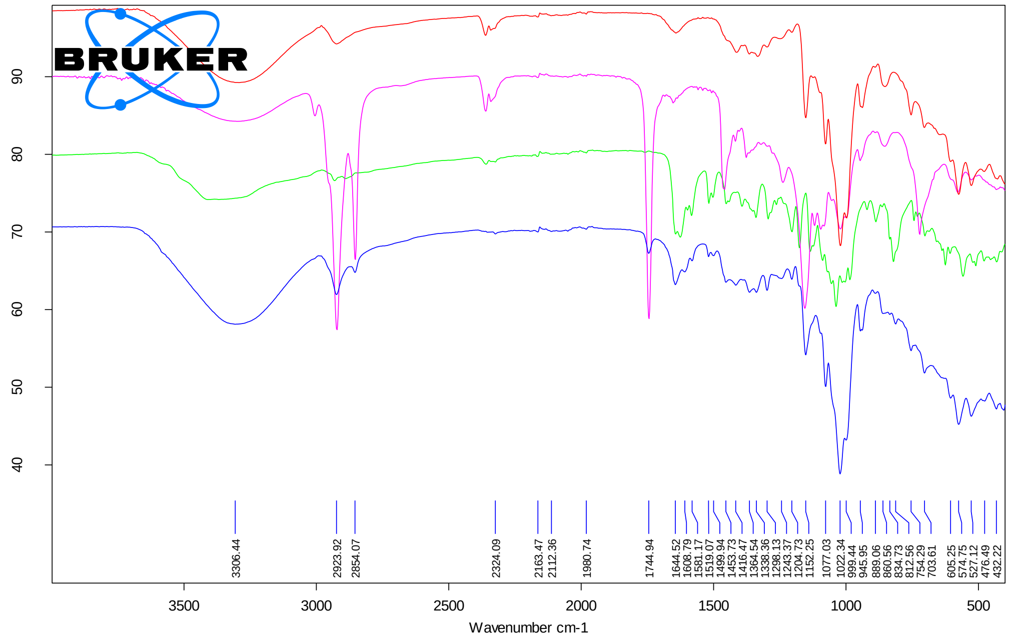

Figure S7: Superposition of the FTIR spectra for the β-cyclodextrin/*Corylus avellana* oil/naringin ternary complex at a 3:1:1 molar ratio (blue), β-cyclodextrin hydrate (red), *C. avellana* oil (pink), and naringin (green).

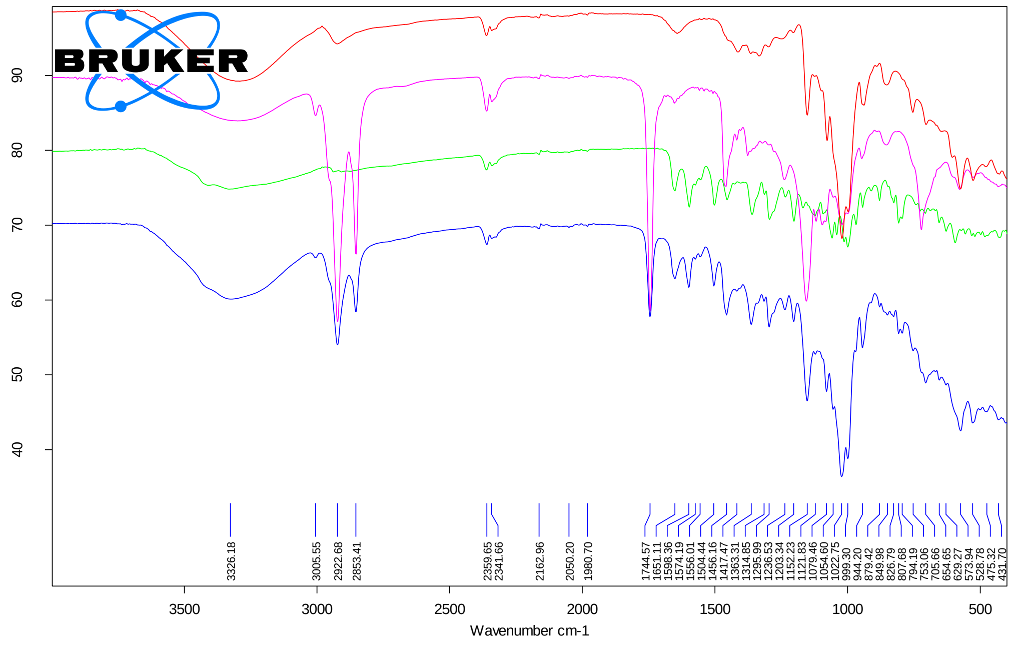

Figure S8: Superposition of the FTIR spectra for the β-cyclodextrin/*Corylus avellana* oil/rutin ternary complex at a 1:1:1 molar ratio (blue), β-cyclodextrin hydrate (red), *C. avellana* oil (pink), and rutin (green).

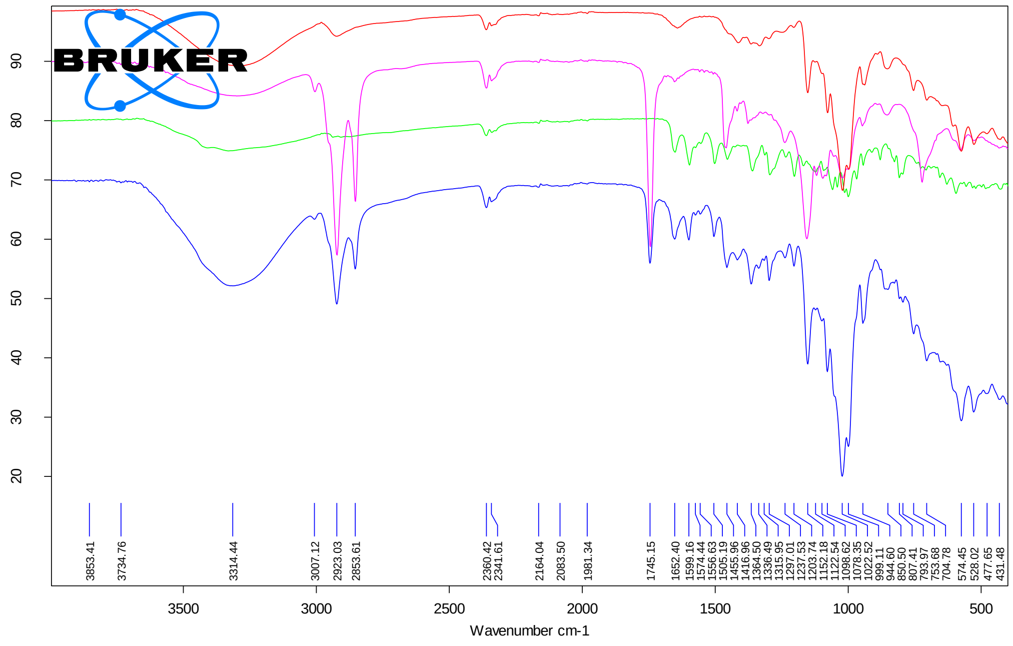

Figure S9: Superposition of the FTIR spectra for the β-cyclodextrin/*Corylus avellana* oil/rutin ternary complex at a 3:1:1 molar ratio (blue), β-cyclodextrin hydrate (red), *C. avellana* oil (pink), and rutin (green).

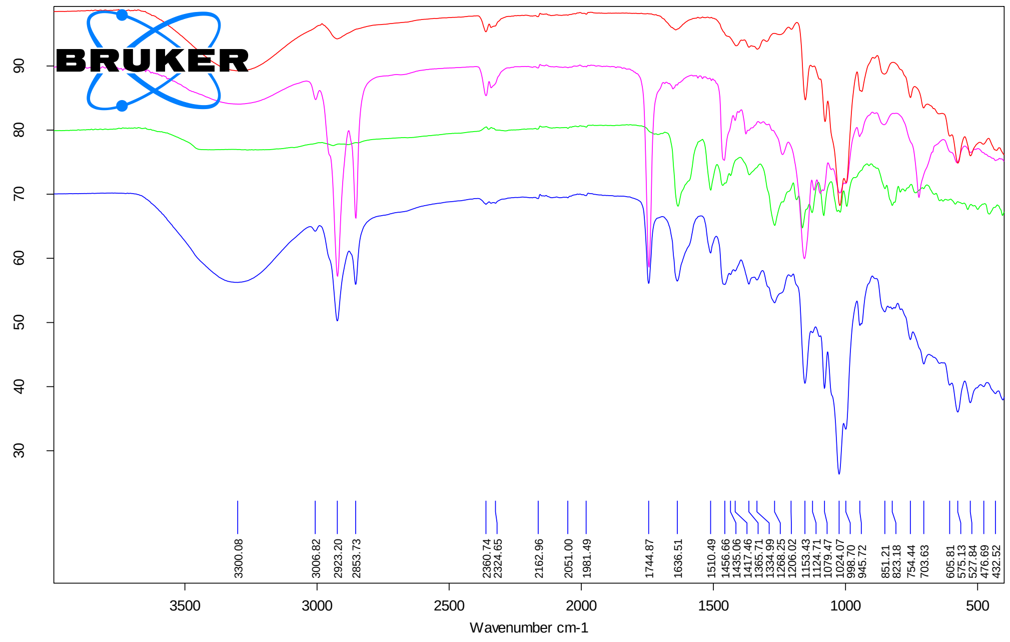

Figure S10: Superposition of the FTIR spectra for the β-cyclodextrin/*Corylus avellana* oil/silymarin ternary complex at a 1:1:1 molar ratio (blue), β-cyclodextrin hydrate (red), *C. avellana* oil (pink), and silymarin (green).

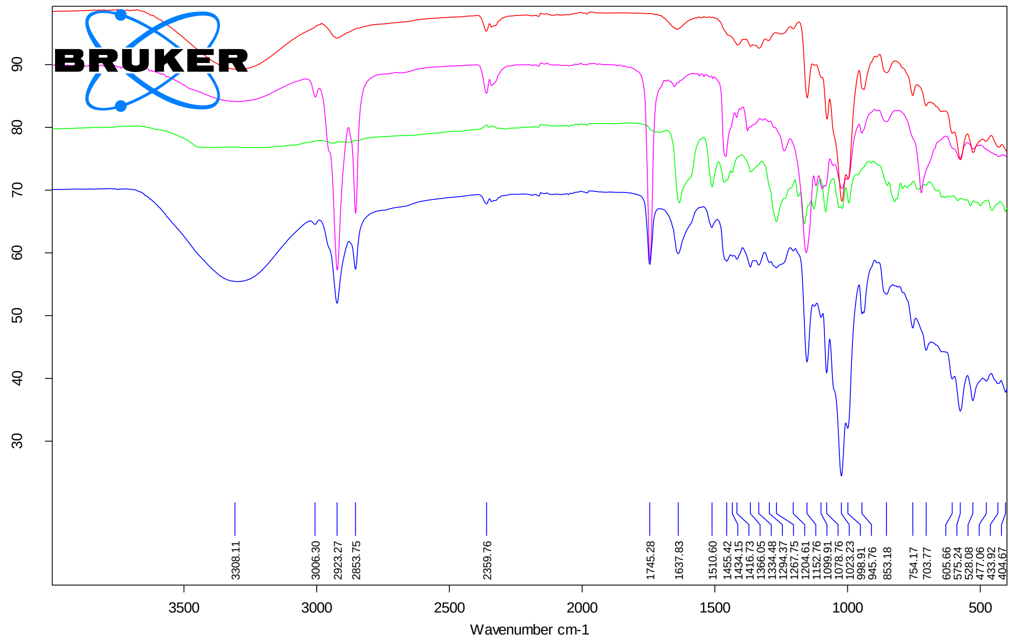

Figure S11: Superposition of the FTIR spectra for the β-cyclodextrin/*Corylus avellana* oil/silymarin ternary complex at a 3:1:1 molar ratio (blue), β-cyclodextrin hydrate (red), *C. avellana* oil (pink), and silymarin (green).

Table S4: FTIR band assignments for β-CD hydrate (mean(±SD) of triplicate determinations).

Wavenumber (cm^{-1})	Band assignment
3301.6(±8.5)	ν_{OH}, stretching vibration of the O-H groups in β-CD and water
2924.8(±1.4)	ν^{as}_{CH}, stretching vibrations of the C-H groups
1643.3(±1.8)	δ_{OH}, bending vibrations of the O-H groups
1451(±0.2)	δ_{CH2}, symmetric bending vibrations of the CH_2 groups
1413.7(±0.8)	δ_{OH}, in-plane bending vibrations of the O-H groups
1364.9(±0.3)	δ_{CH2}, asymmetric bending vibrations of the CH_2 groups
1333.8(±0.2)	δ_{OH}, bending vibrations of the O-H groups
1297.9(±0.4)	δ_{CH}, in-plane bending vibrations of the C-H groups (*tentative*)
1248(±0.9)	δ_{CH}, in-plane bending vibrations of the C-H groups (*tentative*)
1204.7(±0.3)	δ_{CH}, in-plane bending vibrations of the C-H groups (*tentative*)
1152.1(±0.1)	ν^{s}_{COC}, stretching vibrations of the C-O-C groups in glucosydic moieties
1077.2(±0.1)	ν_{CC}, stretching vibrations of the C-C groups
1020.9(±0.3)	ν_{CO}, stretching vibrations of the C-O groups
997.7(±0.2)	ν_{CO}, stretching vibrations of the C-O groups (*tentative*)
939.2(±1.8)	ν_{rgCH}, stretching vibrations of the C-H groups from the β-cyclodextrin ring
852.9(±0.8)	δ_{CCH}, bending vibrations of the C-C-H groups (the α-type glycosidic bonds)
754.4(±0.3)	δ_{CH}, bending vibrations of the C-H groups (*tentative*)
704(±0.7)	δ_{CH}, bending vibrations of the C-H groups (*tentative*)
648.1(±0.9)	*not assigned*
574.2(±0.8)	δ_{OCC}, bending vibrations of the O-C-C groups (*tentative*)
526.3(±1.3)	ν_{CC}, stretching vibrations of the C-C groups (*tentative*)

Table S5: FTIR band assignments for the hazelnut (*Corylus avellana* L.) oil (mean(±SD) of triplicate determinations).

Wavenumber (cm^{-1})	Band assignment
3287.8(±10)	ν_{OH}, stretching vibrations of the O-H groups from the free fatty acids and water
3005(±0.2)	$\nu^{s}_{=CH}$, symmetric stretching vibrations of the =CH groups
2952.5(±0.3)	ν^{as}_{CH}, asymmetric stretching vibrations of the CH groups
2922.5(±0)	ν^{as}_{CH}, symmetric stretching vibrations of the CH groups
2853.2(±0)	ν^{s}_{CH}, symmetric stretching vibrations of the CH groups
1744(±0)	$\nu_{estC=O}$, stretching vibrations of the esteric C=O groups in triglycerides
1710.3(±0.7)	$\nu_{faC=O}$, stretching vibrations of the C=O groups in free fatty acids (shoulder)
1652.7(±0.3)	$\nu_{cC=C}$, stretching vibrations of the *cis*RHC=CHR’ groups
1458.7(±0.2)	$\delta_{CH2/3}$, deformation vibrations of the CH_2 and CH_3 groups

1417.6(±0.1)	$\delta_{rk,=CH}$, rocking vibrations of the =C–H groups in *cis*RHC=CHR’
1376.7(±0)	δ_{CH2}, bending vibrations of the CH_2 groups
1236.8(±1.3)	δ_{CH2}, bending vibrations of the CH_2 groups
1158.1(±2.3)	δ_{CH2}, bending vibrations of the CH_2 groups
1094.7(±0.7)	ν_{CO}, stretching vibrations of the C-O groups
1027.9(±5.7)	ν_{CO}, stretching vibrations of the C-O groups
956.7(±8.7)	$\delta_{tC=C}$, bending vibrations of the C=C groups in *trans*RHC=CHR’
722(±0.1)	δ_{opCH}, out-of-plane deformation vibrations in the C-H groups

Table S6: FTIR band assignments for hesperidin and the corresponding β-cyclodextrin/hazelnut oil/hesperidin 1:1:1 and 3:1:1 ternary complexes (codes “X1H and X3H); bands associated to β-CD or hazelnut oil are also specified; wavenumbers (cm^{-1}) are expressed as mean(±SD) of triplicate determinations for hesperidin and duplicate determinations for the ternary complexes.

Wavenumber (cm^{-1})			Band assignment
Hesperidin	X1H	X3H	
3540.5(±1)	–	–	ν_{OH}, stretching vibrations of the O-H groups (phenolic, glycosidic, water)
3467.5(±1.3)	–	–	ν_{OH}, stretching vibrations of the O-H groups (phenolic, glycosidic, water)
3411.3(±0.5)	3312.1(±2.8)	3306.2(±2.1)	ν_{OH}, stretching vibrations of the O-H groups (phenolic, glycosidic, water) (also from β-CD)
–	3006.5(±1)	3009.6(±0.8)	$\nu^{s}_{=CH}$, symmetric stretching vibrations of the =CH groups (from hazelnut oil)
2982(±0.5)	–	–	ν^{as}_{CH}, stretching vibrations of the C-H bonds in the aliphatic CH_3/CH groups
2940.8(±0.8)	–	–	ν^{as}_{CH}, stretching vibrations of the C-H bonds in the aliphatic CH_3/CH groups
2914.2(±0.9)	2922.4(±0.1)	2922.6(±0.1)	ν^{as}_{CH}, stretching vibrations of the C-H bonds in the aliphatic CH_3/CH groups (also from hazelnut oil)
2895.6(±0.4)	–	–	ν^{s}_{CH}, stretching vibrations of the C-H bonds in the aliphatic CH_2 groups
–	2853.4(±0)	2853.5(±0.2)	ν^{s}_{CH}, symmetric stretching vibrations of the CH groups (from hazelnut oil)
–	1745(±0.1)	1745(±0.1)	$\nu_{estC=O}$, stretching vibrations of the esteric C=O groups in triglycerides (from hazelnut oil)
1644.8(±0.5)	1647.2(±0.1)	1647.2(±0.1)	$\nu^{as}_{C=O/C=C}$, asymmetric stretching vibrations of the C=O/C=C groups
1604.4(±0.1)	1605.9(±0.1)	1606.8(±0.3)	$\nu_{CC}/\delta_{arC\#C}$, stretching vibrations of the C-C group in the ring C / bending vibrations of the aromatic C#C groups
1518.3(±0.6)	1519.7(±0.1)	1519.7(±0.1)	$\delta_{arC\#C}$, bending vibrations of the aromatic C#C groups
1504.1(±0.3)	1505.8(±0.7)	1505.1(±1.2)	ν_{CC}, stretching of C-C group in the ring C
1467.5(±1.1)	1457.6(±0.7)	1456.5(±0.3)	δ_{CH3}, asymmetric bending vibrations of the CH_3 groups
1442.5(±0.4)	1445(±0.3)	1446.5(±0.1)	δ_{CH3}, asymmetric bending vibrations of the CH_3 groups
–	1416.6(±0.5)	1416.1(±0.2)	δ_{OH}, in-plane bending vibrations of the O-H groups (from β-CD)
1404(±1)	–	–	$\delta_{CH3}/\delta_{HOC}$, symmetric bending vibrations of the CH_3 groups/in-plane bending vibrations of the H-O-C groups
1356.6(±0.5)	1357(±0)	1365(±0.4)	ν_{CO}, stretching vibrations of the C-O groups
1339.9(±0.6)	1339.1(±0.3)	1336.9(±0.3)	$\delta_{CH3}/\delta_{OCC}$, symmetric bending vibrations of the CH_3/OCC groups (also from β-CD)
1298.2(±0.2)	1299(±0.1)	1298.9(±0)	$\delta_{CH}/\delta_{OCH}/\nu_{CC}$, in-plane bending vibrations of the C-H/OCH groups / stretching vibrations of the C-C groups
1275.7(±0.3)	1276.8(±0)	1277.3(±0.2)	ν_{CO}, stretching vibrations of the C-O groups (carbohydrates and phenolics)

–	1241.4(±0.1)	1242.5(±0.4)	δ_{CH2}, bending vibrations of the CH_2 groups (from hazelnut oil)
1203.3(±0.6)	1204.4(±0)	1204.6(±0)	ν_{CC}/ν_{CO}, stretching vibrations of the C-C groups in ring B / stretching vibrations of the C-O groups (carbohydrates and phenolics)
1182.4(±0.1)	1182.3(±0.1)	1182.3(±0.2)	$\nu_{CO}/\delta_{HCC/HOC}/\nu_{CC}$, stretching vibrations of the C-O groups (carbohydrates and phenolics)/in-plane bending vibrations of the HCC or HOC groups / stretching vibrations of the C-C groups in the ring A
–	1153.5(±0.3)	1152.6(±0.1)	ν^{s}_{COC}, stretching vibrations of the C-O-C groups in glucosydic moieties (from β-CD)
1130(±0.1)	1131.9(±0.4)	1130.5(±0.3)	$\nu_{CO}/\delta_{CCH}/\tau_{CH2}$, stretching vibrations of the C-O groups/ bending vibrations of the C-C-H groups/"twisting" bending vibrations of the CH_2 groups
1093.9(±0.2)	1095(±0.1)	1096.5(±0.3)	ν_{CC}, stretching vibrations of the C-C groups
–	1077.9(±0.2)	1077.4(±0)	ν_{CC}, stretching vibrations of the C-C groups (from β-CD)
1065.5(±1.2)	–	–	ν_{CO}, stretching vibrations of the C-O groups
1053.3(±2.7)	1049.5(±0.3)	1050.4(±0.6)	ν_{CO}, stretching vibrations of the C-O groups
1030.8(±2.3)	–	–	ν_{CO}/ν_{CC}, stretching vibrations of the C-O/C-C groups in the ring B
–	1023.9(±0.5)	1022.3(±0.2)	ν_{CO}, stretching vibrations of the C-O groups (from β-CD)
1010.5(±1.5)	–	–	ν_{CO}/ν_{CC}, stretching vibrations of the C-O/C-C groups (*tentative*)
971.7(±0.9)	–	–	ν_{OC}, stretching vibrations of the O-C groups
–	946.1(±0.1)	946(±0)	ν_{rgCH}, stretching vibrations of the C-H groups from the β-cyclodextrin ring (from β-CD)
911.5(±0.6)	911.6(±0.1)	911.7(±0.6)	τ_{HCCC}, "twisting" bending vibrations of the H-C-C-C groups
–	861.8(±0.2)	861.9(±0.6)	δ_{CCH}, bending vibrations of the C-C-H groups (the α-type glycosidic bonds) (from β-CD)
–	847.6(±0)	848.1(±0)	δ_{CCH}, bending vibrations of the C-C-H groups (the α-type glycosidic bonds) (from β-CD) (*tentative*)
814.1(±0.8)	815.2(±0)	815(±0.1)	δ_{CH}, out-of-plane bending vibrations of the C-H groups
741.5(±1.4)	743.1(±0)	743.5(±0)	τ_{COH}, "twisting" bending vibrations of the C-O-H groups
–	576.1(±0.1)	575.3(±0.4)	δ_{OCC}, bending vibrations of the O-C-C groups (from β-CD) (*tentative*)
–	526.6(±0.2)	527.4(±0.2)	ν_{CC}, stretching vibrations of the C-C groups (from β-CD) (*tentative*)

Table S7: FTIR band assignments for naringin and the corresponding β-cyclodextrin/hazelnut oil/naringin 1:1:1 and 3:1:1 ternary complexes (codes "X1N and X3N); bands associated to β-CD or hazelnut oil are also specified; wavenumbers (cm^{-1}) are expressed as mean(±SD) of triplicate determinations for naringin and duplicate determinations for the ternary complexes (* observed in one duplicate).

	Wavenumber (cm^{-1})		Band assignment
Naringin	X1N	X3N	
3405.3(±4)	3321(±5.2)	3295.8(±15)	ν_{OH}, stretching vibrations of the O-H groups (phenolic, glycosidic, water) (also from β-CD)
–	3006.4(±0.6)	3008.8(±0.8)	ν_{OH}, stretching vibrations of the O-H groups (phenolic, glycosidic, water)
2930.8(±0.6)	2923.1(±0.2)	2923.6(±0.4)	ν^{as}_{CH}, stretching vibrations of the C-H bonds in the aliphatic CH_3/CH groups (also from hazelnut oil)
2889.9(±0.2)	–	–	ν^{s}_{CH}, stretching vibrations of the C-H bonds in the aliphatic CH_2 groups
–	2853.5(±0.1)	2853.7(±0.6)	ν^{s}_{CH}, symmetric stretching vibrations of the CH groups (from hazelnut oil)
–	1745(±0.4)	1743.6(±1.9)	$\nu_{estC=O}$, stretching vibrations of the esteric C=O groups in triglycerides (from hazelnut oil)

1643.5(±0.6)	1644.8(±0.1)	1643.7(±1.1)	$\nu^{as}_{C=O/C=C}$, asymmetric stretching vibrations of the C=O/C=C groups
1625.8(±0.1)	1606.6(±0.1)	1608.8*	$\nu_{CC}/\delta_{arC\#C}$, stretching vibrations of the C-C group in the ring C / bending vibrations of the aromatic C#C groups
1583.2(±0.1)	–	–	$\nu_{CC}/\delta_{arC\#C}$, stretching vibrations of the C-C group in the ring C / bending vibrations of the aromatic C#C groups
1518.1(±0.1)	1519(±0)	1519(±0)	$\delta_{arC\#C}$, bending vibrations of the aromatic C#C groups
1502.6(±0.1)	1500.5(±0.2)	1501.3(±2)	ν_{CC}, stretching of C-C group in the ring C
1452.6(±0.1)	1455.1(±0.5)	1453.4(±0.4)	δ_{CH3}, asymmetric bending vibrations of the CH_3 groups
1441.9(±0.1)	1444.3(±0.1)	1444.4*	δ_{CH3}, asymmetric bending vibrations of the CH_3 groups
–	1417.4(±0)	1415.5(±1.4)	δ_{OH}, in-plane bending vibrations of the O-H groups (from β-CD)
1392.6(±0.1)	–	–	$\delta_{CH3}/\delta_{HOC}$, symmetric bending vibrations of the CH_3 groups/in-plane bending vibrations of the H-O-C groups
1339.9(±0)	1339.7(±0.4)	1339.4(±1.5)	$\delta_{CH3}/\delta_{OCC}$, symmetric bending vibrations of the CH_3/OCC groups (also from β-CD)
1294.1(±0.1)	1297.1(±0)	1297.7(±0.7)	$\delta_{CH}/\delta_{OCH}/\nu_{CC}$, in-plane bending vibrations of the C-H/OCH groups / stretching vibrations of the C-C groups
1204.1(±0.2)	1204.1(±0.2)	1206.3(±2.2)	ν_{CC}/ν_{CO}, stretching vibrations of the C-C groups in ring B / stretching vibrations of the C-O groups (carbohydrates and phenolics)
1175.8(±0.2)	1177.1(±1)	1178.5(±1.8)	$\nu_{CO}/\delta_{HCC/HOC}/\nu_{CC}$, stretching vibrations of the C-O groups (carbohydrates and phenolics)/in-plane bending vibrations of the HCC or HOC groups / stretching vibrations of the C-C groups in the ring A
–	1153.2(±0.5)	1152.6(±0.4)	ν^{s}_{COC}, stretching vibrations of the C-O-C groups in glucosydic moieties (from β-CD)
1135(±0.1)	1141.1(±0.2)	–	$\nu_{CO}/\delta_{CCH}/\tau_{CH2}$, stretching vibrations of the C-O groups/ bending vibrations of the C-C-H groups/“twisting” bending vibrations of the CH_2 groups
1088.2(±0.3)	1095.1(±0.5)	1096.5(±0.2)	ν_{CC}, stretching vibrations of the C-C groups
–	1077.7(±0.7)	1077(±0.1)	ν_{CC}, stretching vibrations of the C-C groups (from β-CD)
1054.9(±0)	1048.1(±0.8)	1051.1(±1.3)	ν_{CO}, stretching vibrations of the C-O groups
1037.4(±0.2)	–	–	ν_{CO}/ν_{CC}, stretching vibrations of the C-O/C-C groups in the ring B
–	1025.7(±0.1)	1022(±0.5)	ν_{CO}, stretching vibrations of the C-O groups (from β-CD)
1014.3(±0.3)	–	–	ν_{CO}/ν_{CC}, stretching vibrations of the C-O/C-C groups (*tentative*)
985.1(±0.1)	–	–	ν_{OC}, stretching vibrations of the O-C groups
–	945.9(±0.1)	946.4(±0.6)	ν_{rgCH}, stretching vibrations of the C-H groups from the β-cyclodextrin ring (from β-CD)
920.8(±0)	911.4(±0.2)	–	τ_{HCCC}, “twisting” bending vibrations of the H-C-C-C groups
–	862.9(±0.4)	858.2(±3.4)	δ_{CCH}, bending vibrations of the C-C-H groups (the α-type glycosidic bonds) (from β-CD)
820.8(±0)	813.5(±0.1)	813.9(±1.8)	δ_{CH}, out-of-plane bending vibrations of the C-H groups
743.4(±0.1)	–	–	τ_{COH}, “twisting” bending vibrations of the C-O-H groups
–	575.5(±0)	574.7(±0)	δ_{OCC}, bending vibrations of the O-C-C groups (from β-CD) (*tentative*)
–	524.2(±3)	526.4(±1)	ν_{CC}, stretching vibrations of the C-C groups (from β-CD) (*tentative*)

Table S8: FTIR band assignments for naringin and the corresponding β-cyclodextrin/hazelnut oil/rutin 1:1:1 and 3:1:1 ternary complexes (codes "X1R and X3R); bands associated to β-CD or hazelnut oil are also specified; wavenumbers (cm^{-1}) are expressed as mean(±SD) of triplicate determinations for rutin and duplicate determinations for the ternary complexes.

	Wavenumber (cm^{-1})		Band assignment
Rutin	X1R	X3R	
3407.4(±2.2)	–	–	ν_{OH}, stretching vibrations of the O-H groups (phenolic, glycosidic, water) (also from β-CD)
3325(±7.3)	3325.1(±1.5)	3310.8(±5.1)	ν_{OH}, stretching vibrations of the O-H groups (phenolic, glycosidic, water)
–	3006.3(±1.1)	3007.5(±0.5)	$\nu^{s}_{=CH}$, symmetric stretching vibrations of the =CH groups (from hazelnut oil)
2938(±0)	–	–	ν^{as}_{CH}, stretching vibrations of the C-H bonds in the aliphatic CH_3/CH groups
2907.3(±0.2)	2922.8(±0.2)	2923.1(±0)	ν^{as}_{CH}, stretching vibrations of the C-H bonds in the aliphatic CH_3/CH groups (also from hazelnut oil)
2875.9(±0.4)	–	–	ν^{s}_{CH}, stretching vibrations of the C-H bonds in the aliphatic CH_2 groups
–	2853.5(±0.2)	2853.7(±0.1)	ν^{s}_{CH}, symmetric stretching vibrations of the CH groups (from hazelnut oil)
–	1744.7(±0.2)	1745.1(±0)	$\nu_{estC=O}$, stretching vibrations of the esteric C=O groups in triglycerides (from hazelnut oil)
1651(±0.1)	1651.7(±0.8)	1651.8(±0.9)	$\nu^{as}_{C=O/C=C}$, asymmetric stretching vibrations of the C=O/C=C groups
1596.8(±0.1)	1598.4(±0.1)	1599.2(±0.1)	$\nu_{CC}/\delta_{arC\#C}$, stretching vibrations of the C-C group in the ring C / bending vibrations of the aromatic C#C groups
1554.1(±0.1)	–	–	$\delta_{arC\#C}$, bending vibrations of the aromatic C#C groups
1502(±0.1)	1504.6(±0.2)	1504.9(±0.4)	ν_{CC}, stretching of C-C group in the ring C
1454.3(±0.2)	1456.2(±0.1)	1455.6(±0.6)	δ_{CH3}, asymmetric bending vibrations of the CH_3 groups
–	1417.7(±0.4)	1416.5(±0.6)	δ_{OH}, in-plane bending vibrations of the O-H groups (from β-CD)
1402.6(±0.4)	–	–	$\delta_{CH3}/\delta_{HOC}$, symmetric bending vibrations of the CH_3 groups/in-plane bending vibrations of the H-O-C groups
1360(±0.1)	1363.3(±0)	1364.6(±0.1)	ν_{CO}, stretching vibrations of the C-O groups
–	1339.4(±0.1)	1336.1(±0.5)	$\delta_{CH3}/\delta_{OCC}$, symmetric bending vibrations of the CH_3/OCC groups (also from β-CD)
1294.7(±0)	1295.9(±0.1)	1296.9(±0.1)	$\delta_{CH}/\delta_{OCH}/\nu_{CC}$, in-plane bending vibrations of the C-H/OCH groups / stretching vibrations of the C-C groups
–	1279.5(±0.7)	1280.5(±0.2)	ν_{CO}, stretching vibrations of the C-O groups (carbohydrates and phenolics)
–	1236.4(±0.1)	1237.8(±0.4)	δ_{CH2}, bending vibrations of the CH_2 groups (from hazelnut oil)
1202.5(±0.1)	1203.5(±0.2)	1203.9(±0.2)	ν_{CC}/ν_{CO}, stretching vibrations of the C-C groups in ring B / stretching vibrations of the C-O groups (carbohydrates and phenolics)
1168.4(±0.2)	–	–	$\nu_{CO}/\delta_{HCC/HOC}/\nu_{CC}$, stretching vibrations of the C-O groups (carbohydrates and phenolics)/in-plane bending vibrations of the HCC or HOC groups / stretching vibrations of the C-C groups in the ring A
–	1152.4(±0.3)	1152.1(±0.1)	ν^{s}_{COC}, stretching vibrations of the C-O-C groups in glucosydic moieties (from β-CD)
–	1121.7(±0.1)	1123(±0.6)	$\nu_{CO}/\nu_{CC}/\delta_{HCC/HOC}/\nu_{CC}$, stretching vibrations of the C-O/C-C groups (phenolics)/ in-plane bending vibrations of the HCC or HOC groups / stretching vibrations of the C-C groups in the ring A
1092.1(±0.3)	–	–	ν_{CC}, stretching vibrations of the C-C groups
–	1079.7(±0.3)	1078.1(±0.3)	ν_{CC}, stretching vibrations of the C-C groups (from β-CD)
1058.6(±0.1)	1054.9(±0.4)	1053.6(±0.3)	ν_{CO}, stretching vibrations of the C-O groups
1041.1(±0)	–	–	ν_{CO}/ν_{CC}, stretching vibrations of the C-O/C-C groups in the ring B
–	1023.2(±0.7)	1022.4(±0.2)	ν_{CO}, stretching vibrations of the C-O groups (from β-CD)
1013.1(±0)	–	–	ν_{CO}/ν_{CC}, stretching vibrations of the C-O/C-C groups (*tentative*)

968(±0.2)	–	–	ν_{OC}, stretching vibrations of the O-C groups
–	944.2(±0)	944.7(±0.1)	ν_{rgCH}, stretching vibrations of the C-H groups from the β-cyclodextrin ring (from β-CD)
911.1(±0.2)	912.1(±0.1)	911.4(±0.1)	τ_{HCCC}, “twisting” bending vibrations of the H-C-C-C groups
–	863.7(±1.3)	862.1(±0)	δ_{CCH}, bending vibrations of the C-C-H groups (the α-type glycosidic bonds) (from β-CD)
–	849.7(±0.4)	850.6(±0.1)	δ_{CCH}, bending vibrations of the C-C-H groups (the α-type glycosidic bonds) (from β-CD) (*tentative*)
807.6(±0)	807.6(±0.2)	807.4(±0)	δ_{CH}, out-of-plane bending vibrations of the C-H groups
742.2(±0.1)	752.8(±0.3)	753.7(±0)	τ_{COH}, “twisting” bending vibrations of the C-O-H groups
–	574.2(±0.4)	574.7(±0.3)	δ_{OCC}, bending vibrations of the O-C-C groups (from β-CD) (*tentative*)
–	528.6(±0.2)	527.9(±0.1)	ν_{CC}, stretching vibrations of the C-C groups (from β-CD) (*tentative*)

Table S9: FTIR band assignments for naringin and the corresponding β-cyclodextrin/hazelnut oil/naringin 1:1:1 and 3:1:1 ternary complexes (codes “X1N and X3N); bands associated to β-CD or hazelnut oil are also specified; wavenumbers (cm^{-1}) are expressed as mean (±SD) of triplicate determinations for naringin and duplicate determinations for the ternary complexes.

	Wavenumber (cm^{-1})		Band assignment
Silymarin	X1S	X3S	
3400.3(±4.2)	3298.1(±6.2)	3302.4(±8.1)	ν_{OH}, stretching vibrations of the O-H groups (phenolic, glycosidic, water) (also from β-CD)
3263.1(±1.3)	–	–	ν_{OH}, stretching vibrations of the O-H groups (phenolic, glycosidic, water)
–	3006.6(±1.6)	3007.6(±1.8)	$\nu^{s}_{=CH}$, symmetric stretching vibrations of the =CH groups (from hazelnut oil)
2938.4(±3)	–	–	ν^{as}_{CH}, stretching vibrations of the C-H bonds in the aliphatic CH_3/CH groups
–	2922.9(±0.2)	2923.3(±0)	ν^{as}_{CH}, stretching vibrations of the C-H bonds in the aliphatic CH_3/CH groups (also from hazelnut oil)
2882(±1.8)	–	–	ν^{s}_{CH}, stretching vibrations of the C-H bonds in the aliphatic CH_2 groups
–	2853.5(±0.1)	2853.7(±0.1)	ν^{s}_{CH}, symmetric stretching vibrations of the CH groups (from hazelnut oil)
–	1744.7(±0)	1745.2(±0.2)	$\nu_{estC=O}$, stretching vibrations of the esteric C=O groups in triglycerides (from hazelnut oil)
1634.1(±0.4)	1637.5(±0.2)	1637.5(±0.5)	$\nu^{as}_{C=O/C=C}$, asymmetric stretching vibrations of the C=O/C=C groups
1509.9(±0.6)	1510.4(±0.2)	1510.1(±0.6)	ν_{CC}, stretching of C-C group in the ring C
1464.3(±0.7)	1455.7(±0.7)	1455.7(±0.4)	δ_{CH3}, asymmetric bending vibrations of the CH_3 groups
–	1416.9(±0.1)	1417.1(±0.6)	δ_{OH}, in-plane bending vibrations of the O-H groups (from β-CD)
1364(±0.4)	1365.9(±0)	1365.7(±0.5)	ν_{CO}, stretching vibrations of the C-O groups
–	1335(±0)	1335(±0.7)	$\delta_{CH3}/\delta_{OCC}$, symmetric bending vibrations of the CH_3/OCC groups (also from β-CD)
1268.1(±0.1)	1268.3(±0.2)	1268(±0.3)	ν_{CO}, stretching vibrations of the C-O groups (carbohydrates and phenolics)
–	1243.5(±0.7)	1242.9(±0.8)	δ_{CH2}, bending vibrations of the CH_2 groups (from hazelnut oil)
–	1204.6(±0.3)	1205.2(±0.4)	ν_{CC}/ν_{CO}, stretching vibrations of the C-C groups in ring B / stretching vibrations of the C-O groups (carbohydrates and phenolics)
1184.7(±0.8)	–	–	$\nu_{CO}/\delta_{HCC/HOC}/\nu_{CC}$, stretching vibrations of the C-O groups (carbohydrates and phenolics)/in-plane bending vibrations of the HCC or HOC groups / stretching vibrations of the C-C groups in the ring A
–	1152.8(±0.1)	1152.8(±0)	ν^{s}_{COC}, stretching vibrations of the C-O-C groups in glucosydic moieties (from β-CD)

1163.3(±0.2)	–	–	$\nu_{CO}/\nu_{CC}/\delta_{HCC/HOC}/\nu_{CC}$, stretching vibrations of the C-O/C-C groups (phenolics)/ in-plane bending vibrations of the HCC or HOC groups / stretching vibrations of the C-C groups in the ring A
1082.5(±0.1)	1078.6(±0.2)	1078.6(±0.3)	ν_{CC}, stretching vibrations of the C-C groups
–	1050.9(±0.2)	1051.4(±0.3)	ν_{CO}, stretching vibrations of the C-O groups
1031.7(±0.2)	–	–	ν_{CO}/ν_{CC}, stretching vibrations of the C-O/C-C groups in the ring B
–	1023.1(±0.3)	1023.1(±0.1)	ν_{CO}, stretching vibrations of the C-O groups (from β-CD)
1020.3(±0.1)	–	–	ν_{CO}/ν_{CC}, stretching vibrations of the C-O/C-C groups (*tentative*)
995.2(±0)	–	–	ν_{OC}, stretching vibrations of the O-C groups
–	946(±0.1)	945.9(±0.2)	ν_{rgCH}, stretching vibrations of the C-H groups from the β-cyclodextrin ring (from β-CD)
–	852.6(±0.2)	852.6(±0.9)	δ_{CCH}, bending vibrations of the C-C-H groups (the α-type glycosidic bonds) (from β-CD) (*tentative*)
819.7(±5.9)	812.7(±0.7)	812.3(±0.8)	δ_{CH}, out-of-plane bending vibrations of the C-H groups
–	575.5(±0.3)	574.9(±0.4)	δ_{OCC}, bending vibrations of the O-C-C groups (from β-CD) (*tentative*)
–	528(±0.2)	528.1(±0)	ν_{CC}, stretching vibrations of the C-C groups (from β-CD) (*tentative*)

3. Fourier transform infrared spectroscopy–principal component analysis (FTIR–PCA) of ternary complexes.

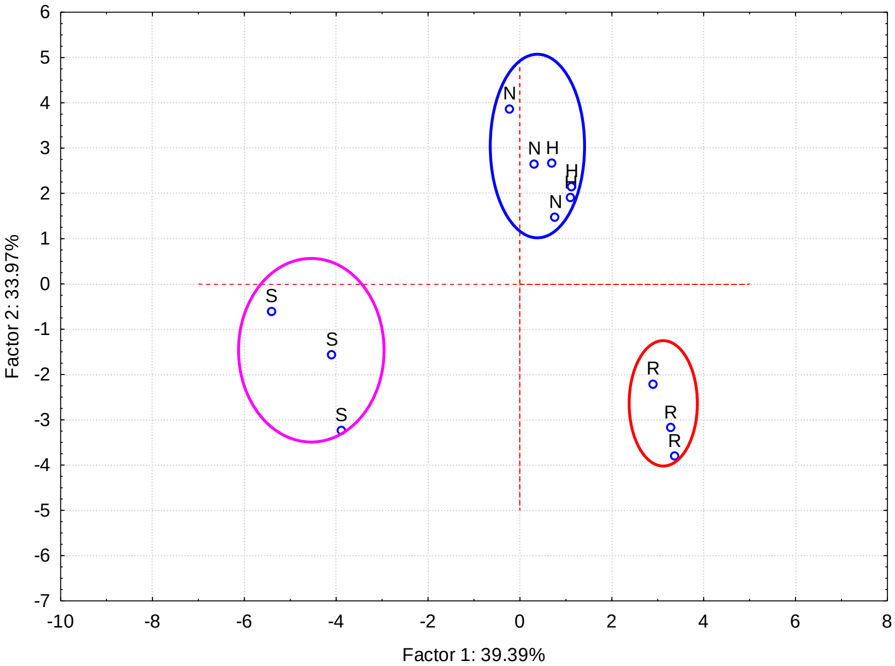

Figure S12: PC_2 *versus* PC_1 scores plot from the FTIR–PCA analysis of the flavonoid glycoside and flavonolignan antioxidants (codes: "H" – hesperidin, "N" – naringin, "R" – rutin and "S" – silymarin); all wavenumber and intensity of the FTIR bands were used as input variables.

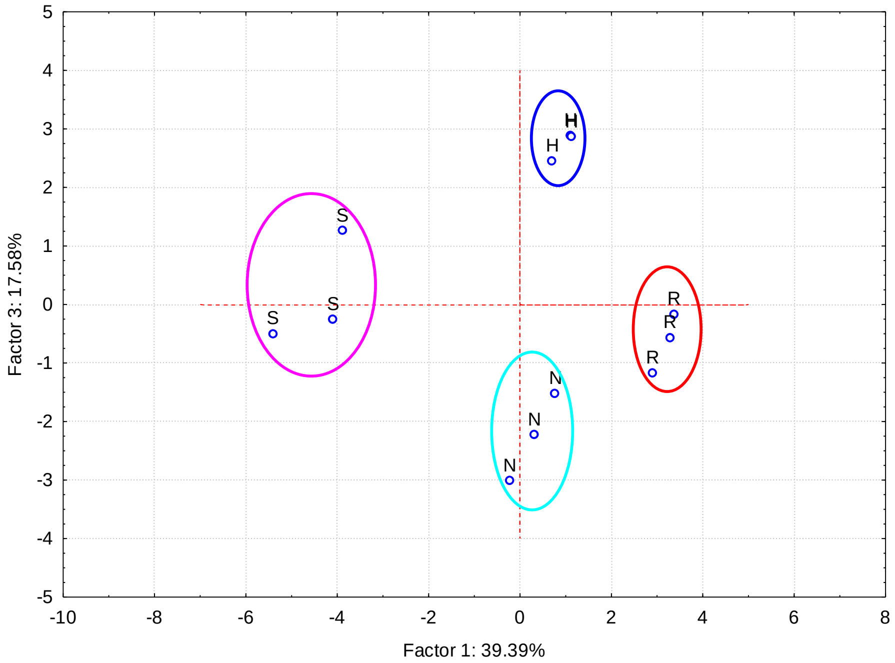

Figure S13: PC_3 *versus* PC_1 scores plot from the FTIR–PCA analysis of the flavonoid glycoside and flavonolignan antioxidants (codes: "H" – hesperidin, "N" – naringin, "R" – rutin and "S" – silymarin); all wavenumber and intensity of the FTIR bands were used as input variables.

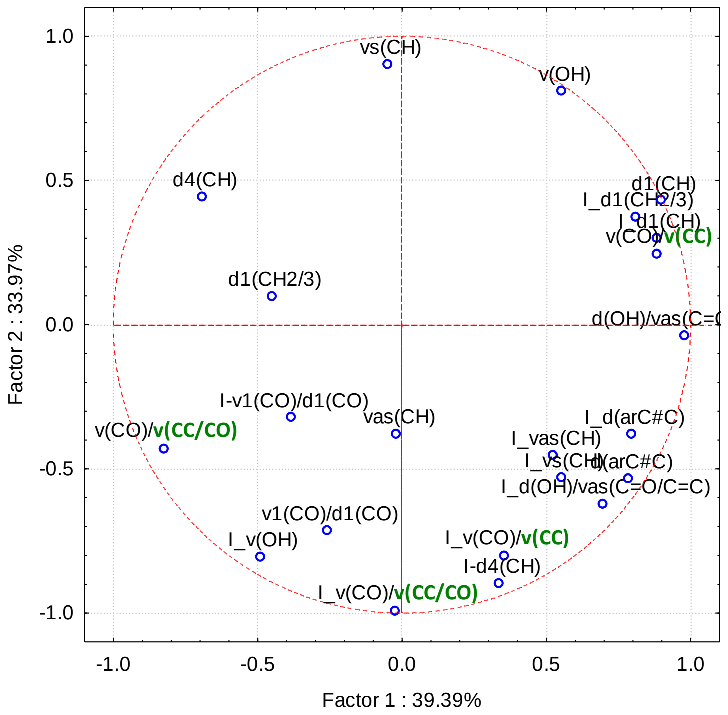

Figure S14: PC_2 *versus* PC_1 loadings plot from the FTIR–PCA analysis of the flavonoid glycoside and flavonolignan antioxidants; all wavenumber and intensity of the FTIR bands were used as input variables (see Table S10 for codes).

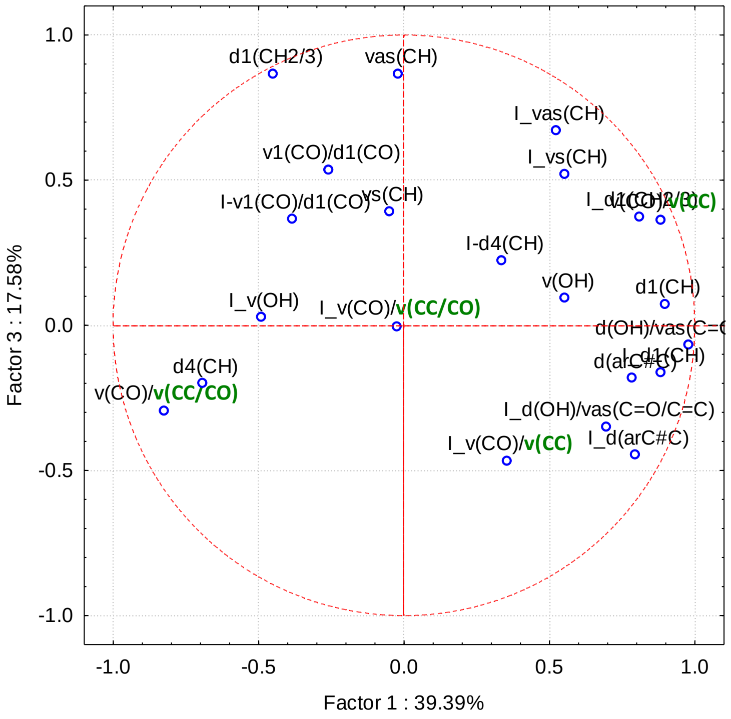

Figure S15: PC_3 *versus* PC_1 loadings plot from the FTIR–PCA analysis of the flavonoid glycoside and flavonolignan antioxidants; all wavenumber and intensity of the FTIR bands were used as input variables (see Table S10 for codes).

Table S10: Factor coordinates (principal components, PCs) of the variables, based on correlations, from the FTIR–PCA analysis of the flavonoid glycoside and flavonolignan antioxidants; all wavenumber (“v” – for stretching vibrations, “d” – for bending vibrations) and intensity (designed as “I_v/d”) of the FTIR bands were used as input variables.

	**PC_1**	**PC_2**	**PC_3**
**v(OH)**	0.555	0.809	0.093
**I_v(OH)**	-0.492	-0.805	0.027
**vas(CH)**	-0.019	-0.379	0.866
**I_vas(CH)**	0.524	-0.452	0.670
**vs(CH)**	-0.049	0.902	0.392
**I_vs(CH)**	0.552	-0.531	0.521
**d(OH)/vas(C=O/C=C)**	0.980	-0.039	-0.068
**I_d(OH)/vas(C=O/C=C)**	0.696	-0.621	-0.349
**d(arC#C)**	0.786	-0.533	-0.183
**I_d(arC#C)**	0.797	-0.380	-0.448
**d1(CH2/3)**	-0.450	0.099	0.867
**I_d1(CH2/3)**	0.809	0.374	0.375
**v1(CO)/d1(CO)**	-0.259	-0.714	0.536
**I-v1(CO)/d1(CO)**	-0.383	-0.323	0.365
**d1(CH)**	0.898	0.430	0.073
**I_d1(CH)**	0.883	0.300	-0.163
**v(CO)/v(CC)**	0.882	0.243	0.364
**I_v(CO)/v(CC)**	0.356	-0.801	-0.469
**v(CO)/v(CC/CO)**	-0.826	-0.430	-0.296
**I_v(CO)/v(CC/CO)**	-0.023	-0.992	-0.004
**d4(CH)**	-0.693	0.444	-0.202
**I-d4(CH)**	0.337	-0.897	0.223

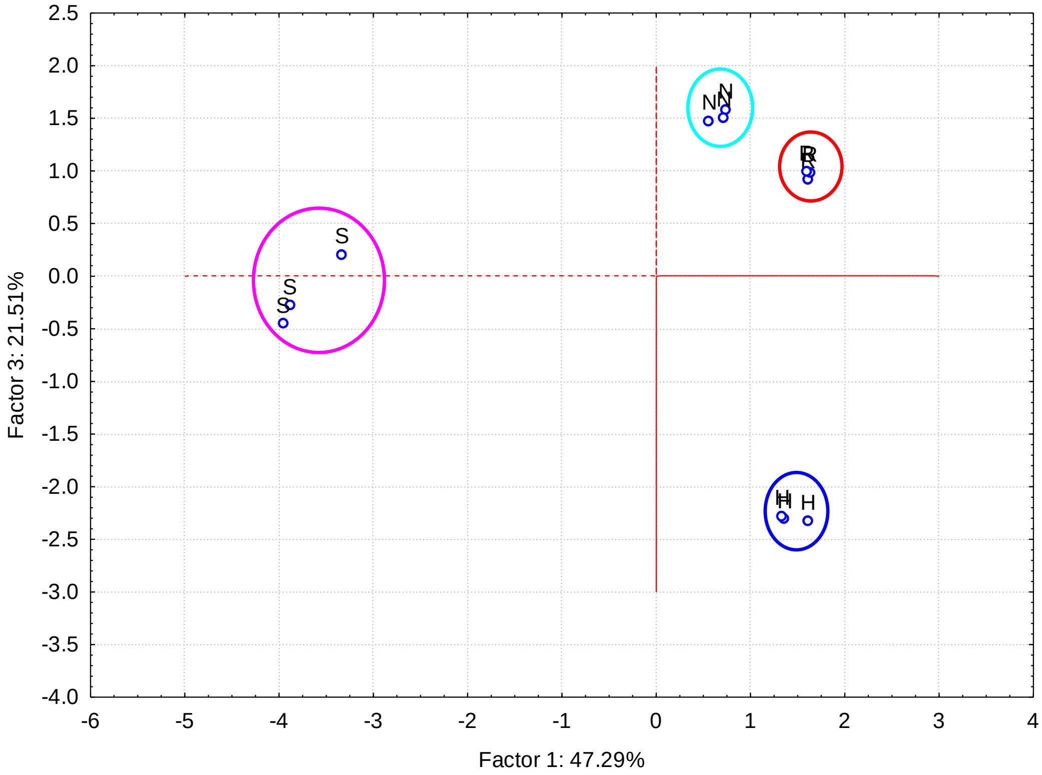

Figure S16: PC_3 *versus* PC_1 scores plot from the FTIR–PCA analysis of the flavonoid glycoside and flavonolignan antioxidants (codes: “H” – hesperidin, “N” – naringin, “R” – rutin and “S” – silymarin); only wavenumbers of the FTIR bands were used as input variables.

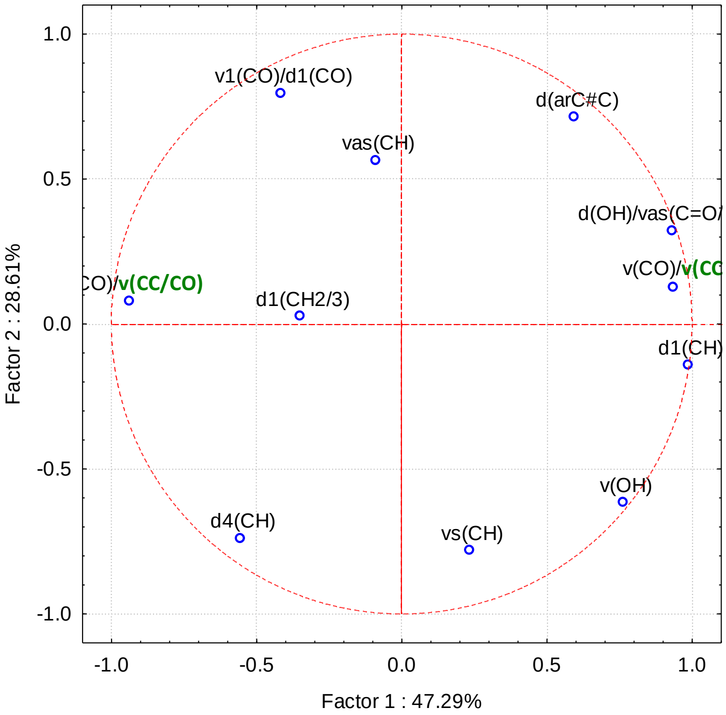

Figure S17: PC_2 *versus* PC_1 loadings plot from the FTIR–PCA analysis of the flavonoid glycoside and flavonolignan antioxidants; only wavenumbers of the FTIR bands were used as input variables (see Table S11 for codes).

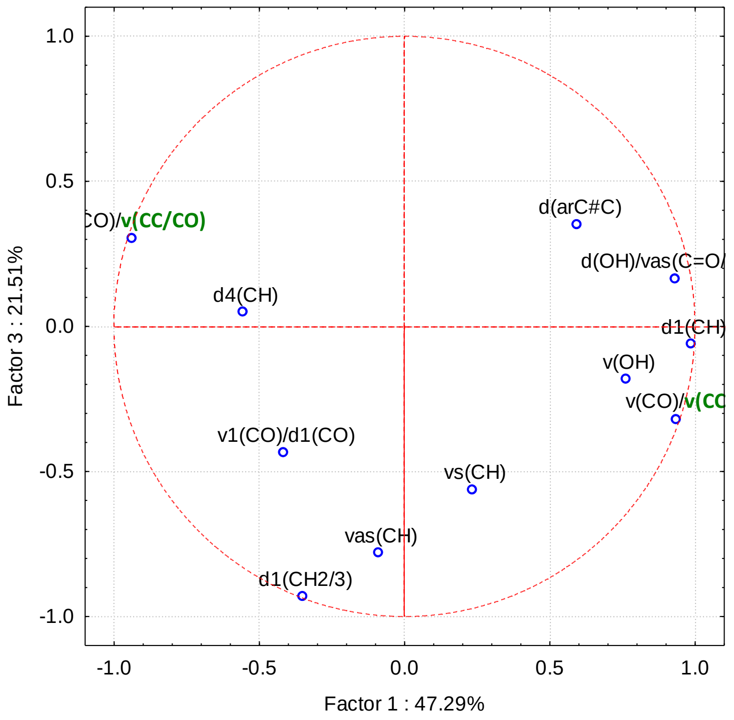

Figure S18: PC_3 *versus* PC_1 loadings plot from the FTIR–PCA analysis of the flavonoid glycoside and flavonolignan antioxidants; only wavenumbers of the FTIR bands were used as input variables (see Table S11 for codes).

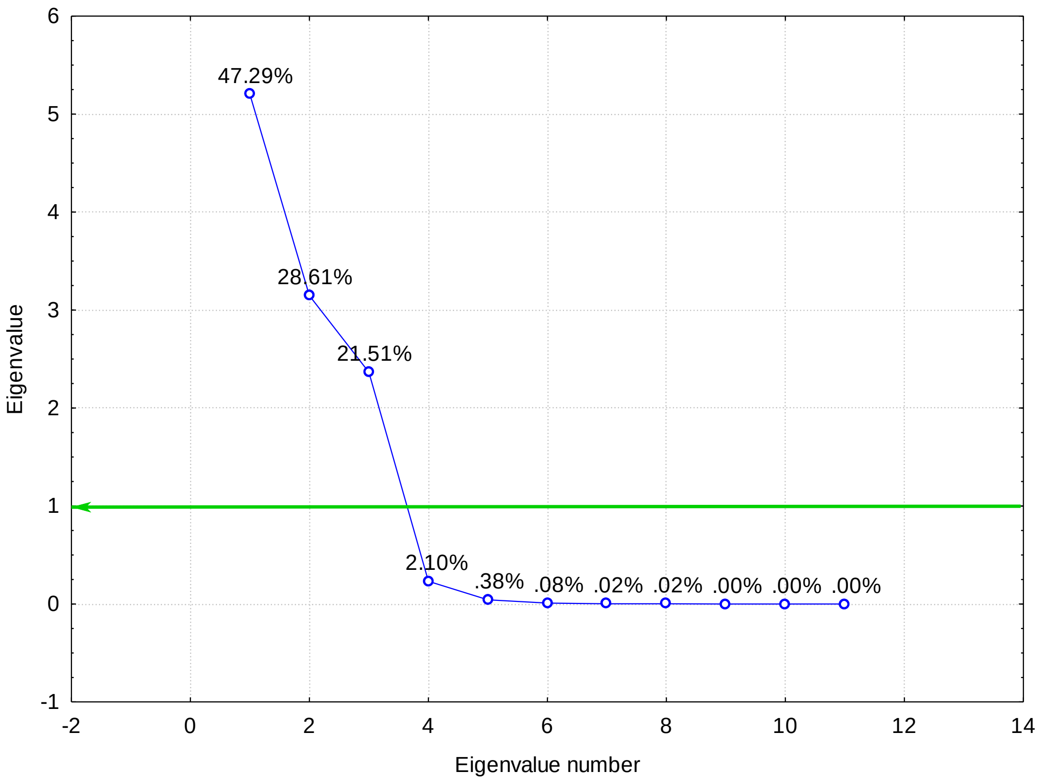

Figure S19: Eigenvalues of the correlation matrix from the FTIR–PCA analysis of the flavonoid glycoside and flavonolignan antioxidants; only wavenumbers of the FTIR bands were used as input variables (see Table S11 for codes); the first three PCs can be retained, which explain 97.41% from the variance of the data.

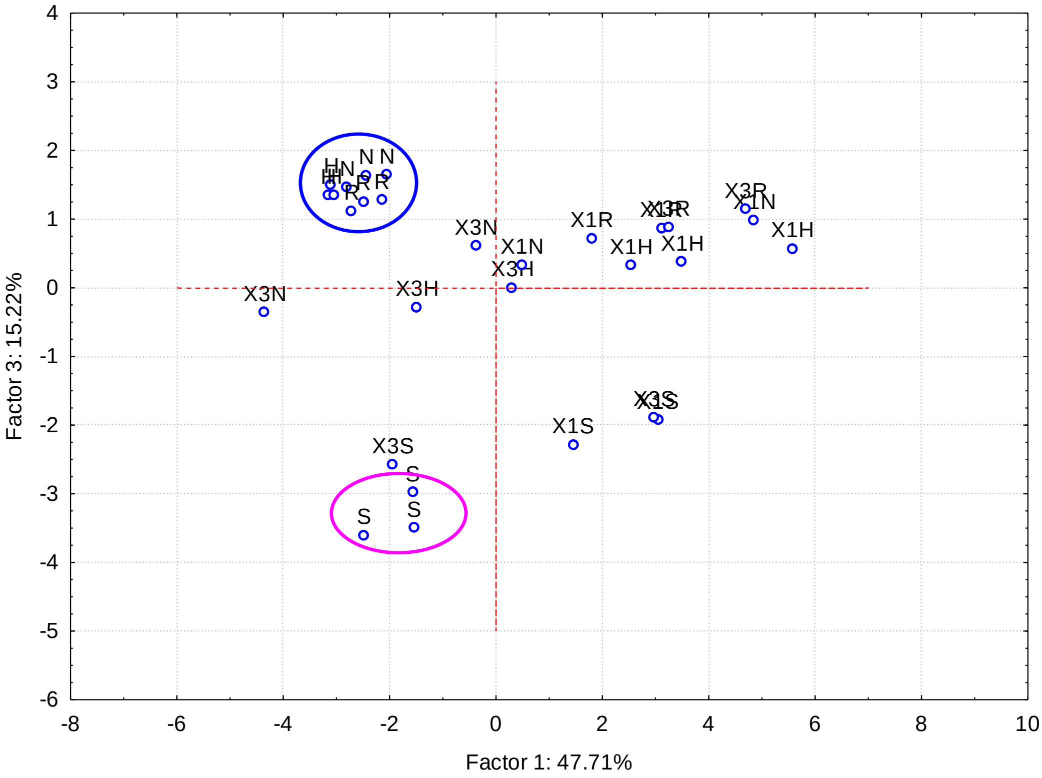

Figure S20: PC_3 *versus* PC_1 scores plot from the FTIR–PCA analysis of the β-CD/hazelnut oil/flavonoid 1:1:1 and 3:1:1 ternary complexes (codes: "X1H/N/R/S" and "X3H/N/R/S") with hesperidin/naringin/rutin/silymarin, respectively) and flavonoids (codes: "H" – hesperidin, "N" – naringin, "R" – rutin and "S" – silymarin); all wavenumber and intensity of the FTIR bands were used as input variables.

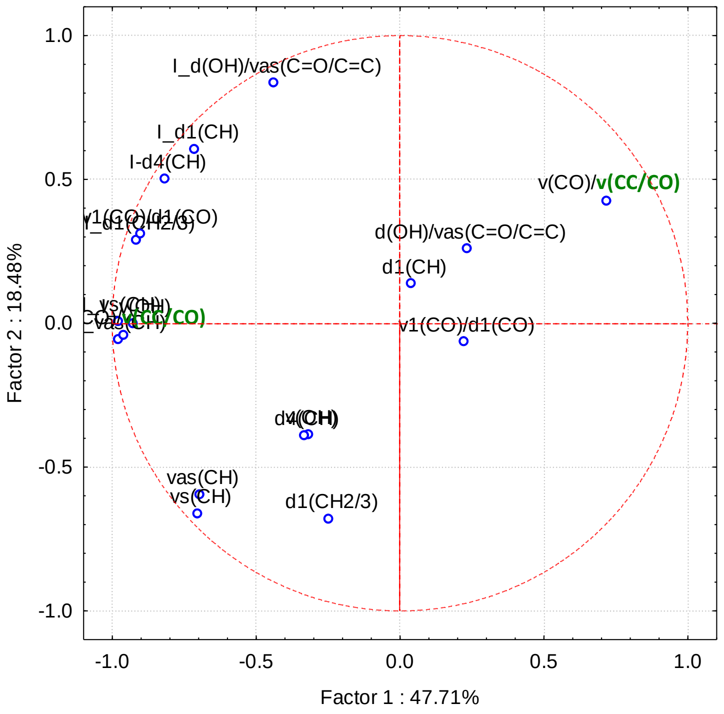

Figure S21: PC_2 *versus* PC_1 loadings plot from the FTIR–PCA analysis of the β-CD/hazelnut oil/flavonoid ternary complexes and flavonoids; all wavenumber and intensity of the FTIR bands were used as input variables (see Table S12 for codes).

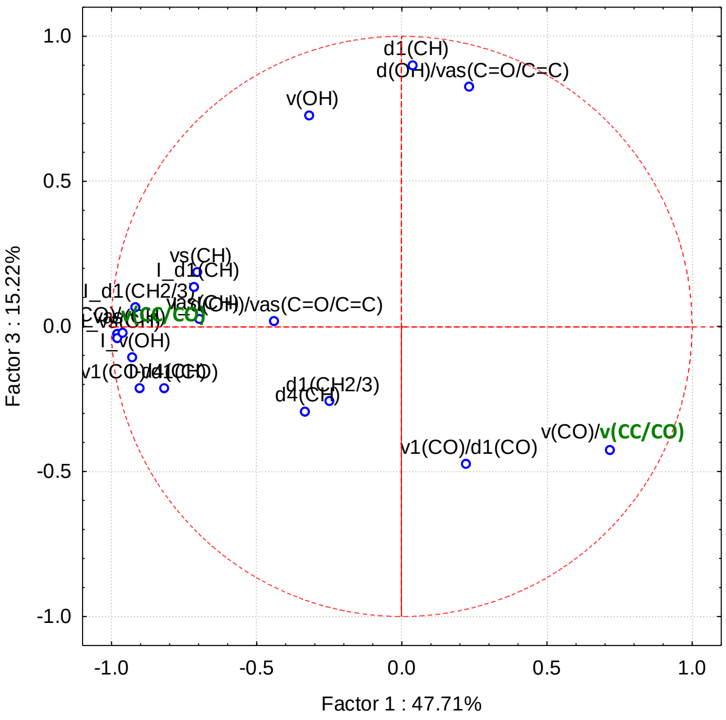

Figure S22: PC_3 *versus* PC_1 loadings plot from the FTIR–PCA analysis of the β-CD/hazelnut oil/flavonoid ternary complexes and flavonoids; all wavenumber and intensity of the FTIR bands were used as input variables (see Table S12 for codes).

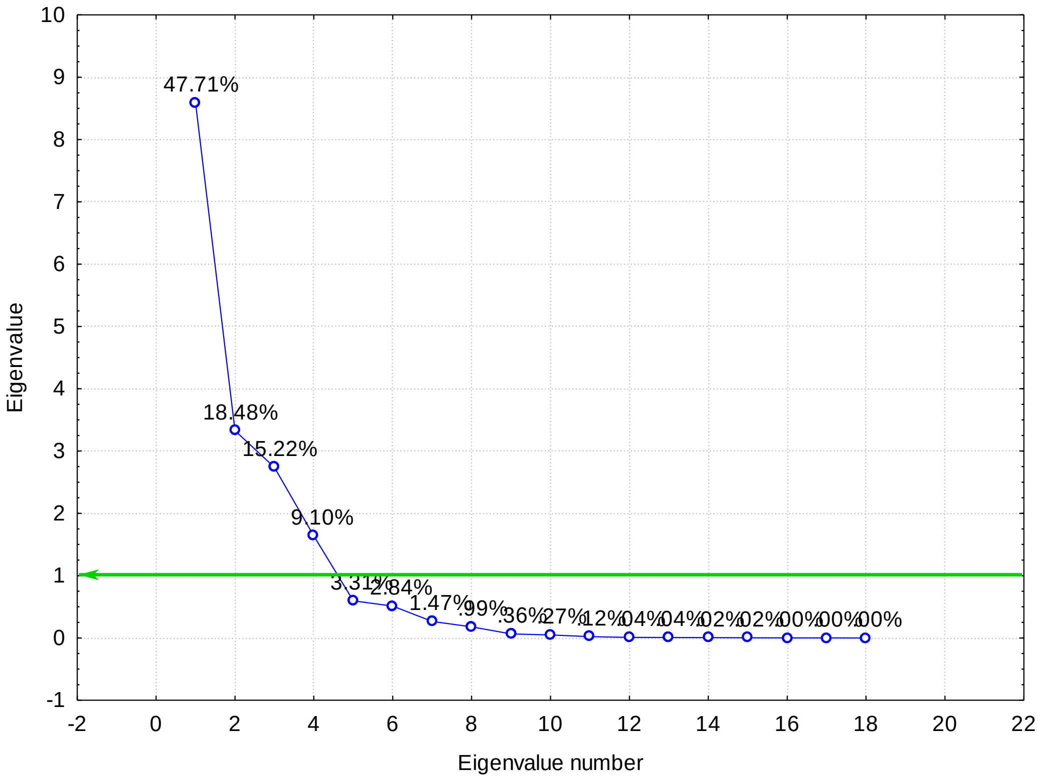

Figure S23: Eigenvalues of the correlation matrix from the FTIR–PCA analysis of the β-CD/hazelnut oil/flavonoid ternary complexes and flavonoids; all wavenumber and intensity of the FTIR bands were used as input variables (see Table S12 for codes); the first four PCs can be retained, which explain 90.51% from the variance of the data.

Table S11: Factor coordinates (principal components, PCs) of the variables, based on correlations, from the FTIR–PCA analysis of the β-CD/hazelnut oil/flavonoid ternary complexes and flavonoids; all wavenumber ("v" – for stretching vibrations, "d" – for bending vibrations) and intensity (designed as "I_v/d") of the FTIR bands were used as input variables.

	PC_1	PC_2	PC_3	PC_4
**v(OH)**	-0.318	-0.387	0.727	0.169
**I_v(OH)**	-0.928	-0.001	-0.109	-0.015
**vas(CH)**	-0.695	-0.595	0.023	-0.305
**I_vas(CH)**	-0.979	-0.056	-0.026	-0.056
**vs(CH)**	-0.702	-0.662	0.185	-0.014
**I_vs(CH)**	-0.979	0.005	-0.042	-0.043
**d(OH)/vas(C=O/C=C)**	0.235	0.258	0.824	-0.372
**I_d(OH)/vas(C=O/C=C)**	-0.437	0.834	0.016	-0.246
**d1(CH2/3)**	-0.247	-0.680	-0.259	-0.298
**I_d1(CH2/3)**	-0.914	0.290	0.063	0.067
**v1(CO)/d1(CO)**	0.222	-0.065	-0.475	-0.758
**I-v1(CO)/d1(CO)**	-0.901	0.310	-0.215	0.146
**d1(CH)**	0.040	0.136	0.899	0.035
**I_d1(CH)**	-0.712	0.605	0.135	0.178
**v(CO)/v(CC/CO)**	0.718	0.426	-0.426	0.223
**I_v(CO)/v(CC/CO)**	-0.961	-0.044	-0.022	-0.075
**d4(CH)**	-0.334	-0.390	-0.294	0.717
**I-d4(CH)**	-0.817	0.501	-0.213	-0.148

Table S12: Factor coordinates (principal components, PCs) of the variables, based on correlations, from the FTIR–PCA analysis of the β-CD/hazelnut oil/flavonoid ternary complexes and flavonoids; only wavenumbers ("v" – for stretching vibrations, "d" – for bending vibrations) of the FTIR bands were used as input variables.

	PC_1	PC_2	PC_3
**v(OH)**	0.683	-0.551	0.141
**vas(CH)**	0.888	0.177	-0.270
**vs(CH)**	0.981	0.048	0.009
**d(OH)/vas(C=O/C=C)**	-0.153	-0.890	-0.370
**d1(CH2/3)**	0.562	0.441	-0.372
**v1(CO)/d1(CO)**	-0.216	0.461	-0.787
**d1(CH)**	0.073	-0.916	0.011
**v(CO)/v(CC/CO)**	-0.904	0.236	0.190
**d4(CH)**	0.411	0.410	0.703
